# Polymers of ε-Caprolactone Using New Copper(II) and Zinc(II) Complexes as Initiators: Synthesis, Characterization and X-Ray Crystal Structures

**DOI:** 10.3390/polym10111239

**Published:** 2018-11-08

**Authors:** Andrés F. Posada, Mario A. Macías, Santiago Movilla, Gian Pietro Miscione, León D. Pérez, John J. Hurtado

**Affiliations:** 1 Departamento de Química, Universidad de los Andes, Carrera 1 No. 18A-12, Bogotá 111711, Colombia; af.posada@uniandes.edu.co (A.F.P.); ma.maciasl@uniandes.edu.co (M.A.M.); s.movilla82@uniandes.edu.co (S.M.); gp.miscione57@uniandes.edu.co (G.P.M.); 2 Grupo de Macromoléculas, Departamento de Química, Universidad Nacional de Colombia, Carrera 45 No 26–85, edificio 451 of. 449, 11001, Bogotá, Colombia; ldperezp@unal.edu.co

**Keywords:** ring-opening polymerization, ε-caprolactone, bis(3,5-dimethylpyrazole) ligands, zinc(II) and copper(II) complexes, crystal structures

## Abstract

Five of six new Zn(II) and Cu(II) complexes were active in the ring-opening polymerization (ROP) of ε-caprolactone (CL) under solvent-free conditions, producing polycaprolactones (PCLs) of high crystallinity with molecular weights between 22,900 and 38,700 g mol^−1^ and decomposition temperatures above 260 °C. ^1^H NMR analysis demonstrated that the PCLs obtained were mainly linear, having hydroxymethylene groups at the chain ends. The results obtained indicated a significant improvement in terms of the ratio of monomer:initiator compared to related Cu(II) and Zn(II) complexes. In addition, the structures of the complexes **1** and **4** were determined by single-crystal X-ray diffraction. The synthesis and full characterization of all complexes are described in this paper.

## 1. Introduction

Currently, the massive disposal of non-eco-friendly polymer packages derived from oil produces a negative environmental impact because they are not biodegradable [1] and tend to accumulate in great amounts worldwide. Polycaprolactone (PCL) is a very important biocompatible and biodegradable polymer that is used in tissue engineering, implants, drug-delivery systems, packaging, and other applications because of its adjustable chemical and mechanical properties [2,3]. PCL can be obtained by the ring-opening polymerization (ROP) of ε-caprolactone (CL) that is initiated or catalyzed by metal complexes [4,5]. Tin catalysts, such as tin octoate, are used industrially for the production of PCL, which requires several purification steps to remove traces of this metal from the biopolymers because of its toxicity, making the polymerization reaction more expensive [6]. Some catalysts also require special handling techniques such as Schlenk lines with complex setups or moisture-free conditions [7,8]. Therefore, the development of new air-stable catalysts with low toxicity for use at moderate reaction temperatures under atmospheric conditions is required. In this study, the authors report on the synthesis, characterization, and application of some air-stable Zn(II) and Cu(II) catalysts that contain a bis(3,5-dimethylpyrazol-1-yl)methane ligand for the polymerization of CL. The ROP was conducted under solvent-free conditions at a complex:monomer ratio of 1:100. The PCL that was obtained exhibited a crystallinity up to 89% and a decomposition temperature above 470 °C [9]. Related Zn(II) and Cu(II) complexes have shown activity in ROP of CL at 110 °C, producing polymers with moderate molecular weights and molecular weight distributions [10,11].

It has been reported that ancillary ligands can modify metal complexes that then favor the polymerization of lactones [12,13]. In some cases, pyrazolyl, triazole, and pyrazole derivatives act as nitrogen-donor ligands and therefore increase the Lewis acidity of the metal to enhance the coordination to the lactone [14,15,16]. Specifically, the combination of nitrogen- and oxygen-donor ligands produce active Zn(II) and Cu(II) complexes for the ROP of ε-caprolactone [13,17].

Consequently, following their interest in pyrazolyl ligands in transition metal complexes for catalysis [18,19,20] and taking into account the work reported by Appavoo et al. [17], the authors synthetized, characterized, and applied neutral Zn(II) and Cu(II) complexes containing 3,5-dimethylpyrazole and benzoic acid ligands with stronger EWG substituents on the N-donor ligands in order to increase their activity for the polymerization of CL.

## 2. Materials and Methods 

The reactants and solvents used in this work were obtained from Alfa Aesar (Ward Hill, MA, USA) and Sigma-Aldrich (Saint Louis, MO, USA), and all of the solvents were dried using 4 Å molecular sieves. Elemental analyses (C, H, and N) were conducted using a Thermo Scientific™ FLASH 2000 CHNS/O Analyzer (Thermo Fisher Scientific, Waltham, MA, USA). Fourier transform infrared (FTIR) spectra were obtained using a Thermo Nicolet NEXUS FTIR spectrophotometer using KBr pellets. Ultraviolet/Visible (UV/vis) spectra were obtained using an Agilent Technologies Cary 100 spectrophotometer (Agilent Technologies, Santa Clara, CA, USA). Melting points were determined using a Mel-Temp^®^ 1101D apparatus (Electrothermal, Staffordshire, UK) in open capillary tubes and were uncorrected. NMR data were obtained using a Bruker Avance 400 spectrometer at 295 K (Bruker, Billerica, MA, USA (400.13 MHz for ^1^H; 100.61 MHz for ^13^C)). ^1^H and ^13^C NMR chemical shifts (δ) were reported in parts per million (ppm) relative to TMS, with the residual solvent peak used as an internal reference using CDCl_3_ (^1^H NMR δ: 7.26 and ^13^C NMR δ: 77.2) and DMSO-d_6_ (^1^H NMR δ: 2.50 and ^13^C NMR δ: 39.5).

High-resolution mass spectrometry (HRMS) of ligands was obtained using an Agilent Technologies Q-TOF 6520 spectrometer (Agilent Technologies, Santa Clara, CA, USA) via electrospray ionization (ESI) in positive ion mode. Thermogravimetric (TG) analyses of the complexes and polymers (polycaprolactones) were obtained using a NETZSCH STA 409 PC/PG (NETZSCH, Selb, Bavaria, Germany) by collecting 8–10 mg of the complexes in a nitrogen atmosphere. The samples were subjected to dynamic heating over a temperature range of 30–700 °C at a heating rate of 10 °C min^−1^. The TG curves were analyzed to obtain the percent mass loss as a function of the temperature.

Gel permeation chromatography (GPC) analysis of the molecular weight (Mn and Mw) and molecular weight distribution (PDI = Mw/Mn) of the PCL was performed using a Viscotek GPCmax VE 2001 chromatograph (Worcestershire, UK) equipped with a HR4E column at 35 °C. THF was used as the eluent (0.5 mL min^−1^) and as the solvent to prepare a 5 mg mL^−1^ sample solution. The sample was filtered through a 0.2 µm syringe filter before the analysis and 100 µL of the sample was injected onto the GPC column. Molecular weights were determined based on polystyrene standards. Differential scanning calorimetry (DSC) analysis of the polycaprolactones was performed using a TA Instruments DSC Q200 (Pittsburgh, PA, USA) in a nitrogen atmosphere (50 mL min^−1^). A 7.4 mg sample was heated from 30 to 150 °C, cooled from 150 to −90 °C, and heated from −90 to 90 °C at a heating rate of 5 °C min^−1^. The crystallinity was determined using the following formula:

$$X_{c}=\frac{\Delta H_{exp}}{\Delta H_{u}^{0}},$$

where $\Delta H_{exp}$ is the area of the melting peak and $\Delta H_{u}^{0}$ is the melting enthalpy of 100% crystalline PCL (136.08 J g^−1^) [21].

### 2.1. Synthesis of Ligands

#### 2.1.1. 4-Bromo-3,5-dimethylpyrazole (**Br-Pz**)

**Br-Pz** was synthesized based on a reported method [22] in which the reaction of 3,5-dimethylpyrazole (**Pz**) (1.0 g, 10.4 mmol) was conducted with a stoichiometric amount of N-bromosuccinimide (1.85 g, 10.4 mmol) in carbon tetrachloride (8 mL) at room temperature (rt) for 2 h with stirring. The reaction mixture was then filtered, washed with water (3 × 10 mL), and dried over magnesium sulfate. The solvent was then removed under reduced pressure to obtain a light yellow solid. Yield: 1.63 g (90%). mp: 124–125 °C, RMN ^1^H (400 MHz, CDCl_3_) δ: 2.25 (singlet [s], 6H, CH_3_), 10.11 (broad [b], 1H, NH) ppm.

#### 2.1.2. 4-Iodo-3,5-dimethylpyrazole (**I-Pz**)

The synthesis of **I-Pz** was conducted using a previously reported procedure [23] that involved deprotonation of **Pz** (1.98 g, 20.6 mmol) at position 4 with Na_2_CO_3_ (1.37 g, 12.9 mmol) in water under reflux, and the subsequent addition of 40 mL of an aqueous solution that contained a mixture of KI and I_2_ at a ratio of 2:1. The mixture was filtered and the solid that was obtained was dissolved in dichloromethane (DCM) and washed with a saturated solution of sodium carbonate (15 mL, ×3). The organic layer was dried over MgSO_4_ and the solvent was removed under reduced pressure to finally isolate the product as a white solid. Yield: 3.77 g (82%). mp: 134–135 °C, RMN ^1^H (400 MHz, CDCl_3_) δ: 2.26 (s, 6H, CH3), 11.71 (b, 1H, NH) ppm.

#### 2.1.3. 4-Nitro-3,5-dimethylpyrazole (**NO_2_-Pz**)

The synthesis of **NO_2_-Pz** has been described by Morgan and Ackerman [24]. The nitration of **Pz** was performed by adding **Pz** (1.0 g, 10.4 mmol) to a mixture of concentrated nitric (1.2 mL, 65%) and sulfuric acids (2 mL, 98%) on an ice bath with stirring at rt for 24 h. The reaction mixture was then neutralized with NaOH (14 mL, 60%) and the white solid precipitate was filtered from the solution and dissolved in DCM. This solution was washed with water and dried over MgSO_4_. The DCM was removed under reduced pressure and a white solid was obtained (**NO_2_-Pz**). Yield: 0.97 g (75%). mp: 123–124 °C, RMN ^1^H (400 MHz, CDCl_3_) δ: 2.61 (s, 6H, CH_3_), 11.74 (b, 1H, NH) ppm.

### 2.2. Synthesis of Complexes

The general methodologies that were used to obtain the complexes of zinc(II) and copper(II) were an adaptation of the synthesis described by Appavoo et al. [17]. Typically, all of the reactions were conducted at rt with stirring for 1 h. In all cases, the metal acetate was added to a methanolic solution of benzoic acid (AB) at a molar ratio of 1:2, respectively. A solution of the corresponding pyrazole derivatives in toluene was then added to the reaction mixture with stirring at rt for 3 h. The molar ratio between the N-donor ligands and the metal acetate was 2:1 in the reactions. Finally, the methanol was removed under reduced pressure and replaced with cold pentane. The complexes precipitated from the solutions after refrigeration at 4.5 °C.

#### 2.2.1. [Zn(C_6_H_5_COO)_2_(**Br-Pz**)_2_] (**1**)

Complex **1** was isolated as a crystalline white solid after the reaction between [Zn(OAc)_2_] (0.11 g, 0.50 mmol), AB (0.12 g, 1.0 mmol), and **Br-Pz** (0.18 g, 1.00 mmol). Yield: 0.30 g (92%). mp: 207–209 °C. NMR ^1^H (400 MHz, CDCl_3_) *δ*: 2.28 (s, 12H, CH_3_), 7.40 (triplet [t], 4H, CH), 7.48 (t, 2H, CH), and 8.12 (doublet [d], 4H, CH) ppm. NMR ^13^C (400 MHz, (CD_3_)_2_SO) δ: 11.25 (C_methyl-PZ_), 92.85 (C_4-PZ_), 128.92 (C_3,3′-AB_), 129.73 (C_1,2,2′-AB_), 133.19 (C_4-AB_), 141.73 (C_3,3′-PZ_), and 167.80 (C_carbonyl-AB_) ppm. FTIR (KBr, cm^−1^): 3450, 2810, 1612, 1550, 1390, 1175, 1100, 840, 774, 710, 678, and 575. HRMS (FTMS + pESI) *m*/*z*, calcd. for [Zn(Br-Pz)(C_6_H_5_COO^−^)_2_ + NH4]^+^: 498.0001; found: 498.1214. Elem. Anal.: calcd. (%): C, 44.08; H, 3.70; N, 8.57; found (%): C, 44.06; H, 3.70; N, 8.56.

#### 2.2.2. [Cu(C_6_H_5_COO)_2_(**Br-Pz**)_2_] (**2**)

Complex **2** was synthesized by reacting AB (0.12 g, 1,0 mmol) with [Cu(OAc)_2_] (0.10 g, 0.50 mmol) and **Br-Pz** (0.18 g, 1.00 mmol) and was obtained as a light blue crystalline solid. Yield: 0.27 g (83%). mp: 240–242 °C. FTIR (KBr, cm^−1^): 3453, 3180, 2324, 1627, 1543, 1401, 1291, 1169, 1102, 1021, 937, 845, 724, 686, and 573. HRMS (FTMS + pESI) *m*/*z*, calcd.: [Cu(Br-Pz)_2_ + 2H]^+^: 412,9033; found: 412,8887. Elem. Anal.: calcd. (%): C, 44.15; H, 3.70; N, 8.58; found (%): C, 44.13; H, 3.70; N, 8.53. UV/Vis (2.5 × 10^−3^ mM acetonitrile) λ_max_, nm (ε, L mol^−1^ cm^−1^): 231 (26280). 

#### 2.2.3. [Zn(C_6_H_5_COO)_2_(**I-Pz**)_2_] (**3**)

Complex **3** was obtained by reacting [Zn(OAc)_2_] (0.11 g, 0.50 mmol) with **I-Pz** (0.45 g, 1.00 mmol) and AB (0.12 g, 1,0 mmol). It was isolated as a white powder. Yield: 0.32 g (85%). mp: 226–228 °C. NMR ^1^H (400 MHz, (CD_3_)_2_SO) δ: 2.12 (s, 6H, CH_3_), 7.45 (t, 4H, CH), 7.51 (d, 2H, CH), and 7.98 (d, 4H, CH) ppm. NMR ^13^C (400 MHz, (CD_3_)_2_SO) δ: 12.94 (C_methyl-PZ_), 62.59 (C_4-PZ_), 128.82 (C_3,3′-AB_), 129.81 (C_1,2,2′-AB_), 132.76 (C_4-AB_), 145.42 (C_3,3′-PZ_), and 169.09 (C_carbonyl-AB_) ppm. FTIR (KBr, cm^−1^): 3451, 3184, 3108, 2980, 2848, 1602, 1568, 1500, 1357, 1290, 1176, 819, 761, 726, 679, and 560. HRMS (FTMS + pESI) *m*/*z*, calcd.: [Zn(I-Pz)_2_(C_6_H_5_COO^−^)]^+^, 6,288,883; found: 6,288,870. Elem. Anal.: calcd. (%): C, 38.44; H, 3.23; N, 7.47; found (%): C, 38.39; H, 3.20; N, 7.43. 

#### 2.2.4. [Cu(C_6_H_5_COO)_2_(I-Pz)_2_] (**4**)

A crystalline purple solid (complex **4**) was isolated after reacting [Cu(OAc)_2_] (0.10 g, 0.50 mmol) with AB (0.12 g, 1.00 mmol) and **I-Pz** (0.45 g, 1.00 mmol). Yield: 0.36 g (95%). mp:182–184 °C. FTIR (KBr, cm^−1^): 3183, 3091, 2995, 2862, 1646, 1607, 1551, 1398, 1305, 1177, 1084, 1030, 848, 725, 687, and 572. HRMS (FTMS + pESI) *m*/*z*, calcd.: [Cu(I-Pz)_2_^•^]^+^, 5,068,604; found: 5,068,689. Elem. Anal.: calcd. (%): C, 38.49; H, 3.23; N, 7.48; found (%): C, 38.48; H, 3.23; N, 7.47. UV/Vis (5.0 × 10^−3^ mM acetonitrile) λ_max_, nm (ε, L mol^−1^ cm^−1^): 228 (19420). 

#### 2.2.5. [Zn(C_6_H_5_COO)_2_(**NO_2_-Pz**)_2_] (**5**)

Complex **5** was isolated as a white powder by reacting [Zn(OAc)_2_] (0.11 g, 0.50 mmol) with AB (0.12 g, 1.00 mmol) and **NO_2_-Pz** (0.14 g, 1.00 mmol). Yield: 0.22 g (74%). mp**:** 291–293 °C. NMR ^1^H (400 MHz, (CD_3_)_2_SO) *δ*: 2.12 (s, 6H, CH_3_), 7.40 (t, 4H, CH), 7.48 (t, 2H, CH), and 8.12 (d, 4H, CH) ppm. NMR ^13^C (400 MHz, (CD_3_)_2_SO) *δ*: 13.04 (C_methyl-PZ_), 128.74 (C_3,3′-AB_), 129.68 (C_1,2,2′-AB_), 131.21 (C_4-PZ_), 133.03 (C_4-AB_), 143.80 (C_3,3′-PZ_), and 167.82 (C_carbonyl-AB_) ppm. FTIR (KBr, cm^−1^): 3403, 3001, 2838, 1550, 1423, 1351, 1182, 1041, 999, 842, 775, 699, 612, and 588. HRMS (FTMS + pESI) *m*/*z*, calcd.: [Zn(NO_2_^−^Pz)_2_ + 2 NH_4_]^2+^, 191,0522; found: 191,0806. Elem. Anal.: calcd. (%): C, 49.02; H, 4.11; N, 14.29; found (%): C, 49.01; H, 4.10; N, 14.27. 

#### 2.2.6. [Cu(C_6_H_5_COO)_2_(**NO_2_-Pz**)_2_] (**6**)

Complex **6** was synthesized by reacting [Cu(OAc)_2_] (0.10 g, 0.50 mmol) with AB (0.12 g, 1.00 mmol) and **NO_2_-Pz** (0.14 g, 1.00 mmol). This compound was isolated as a green powder. Yield: 0.21 g (71%). mp: 258–260 °C. FTIR (KBr, cm^−1^): 3279, 3057, 1637, 1576, 1502, 1400, 1354, 1178, 1062, 1035, 836, 720, 682, and 569. ESI-MS *m*/*z*: calcd.: [Cu(NO_2_^−^Pz) + 2H ]^2+^, 1,035,029; found: 1,039,567. Elem. Anal.: calcd. (%): C, 49.10; H, 4.12; N, 14.31; found (%): C, 49.10; H, 4.10; N, 14.26. UV/Vis (2.5 × 10^−3^ mM acetonitrile) λ_max_, nm (ε, L mol^−1^ cm^−1^): 285 (17280). 

### 2.3. X-ray Structural Determination

The collection of the crystallographic data and refinement details are given in Table 5. In order to measure the X-ray intensities at 298(2) K, an Agilent SuperNova, Dual, Cu at zero, Atlas four-circle diffractometer equipped with a Charge Coupled Device (CCD) plate detector was used (Mo*K*α radiation *λ* = 0.71073 Å). CrysAlisPro software package (Agilent Technologies, Santa Clara, CA, USA, version 1.171.37.35) was used to integrate the collected frames and to correct absorption effects. The crystal structures were initially solved using an iterative algorithm [25] and subsequently completed by difference Fourier maps. The atoms, different from hydrogen, were refined anisotropically, whereas the hydrogen atoms were generated geometrically, placed in their calculated positions (C–H = 0.93–0.96 Å; N–H = 0.86 Å), and included as riding contributions with isotropic displacement parameters set at 1.2–1.5 times the *U_eq_* value of the parent atom. The refinement of the structures was carried out using the SHELXL2014 program [26]. The graphic material was prepared using Mercury 3.8 software [27].

### 2.4. Polymerization of ε-Caprolactone

Polymerization of ε-caprolactone using the Cu(II) and Zn(II) complexes (**1**–**6**) was performed using a [monomer]:[initiator] ([M]/[I]) molar ratio of 100:1 in all of the experiments. CL (0.01 mol, 1.14 g) was added to glass-tube reactors under solvent-free conditions with magnetic stirring at 110 °C. The polymers were purified following a previously reported procedure from the literature and characterized by physico-chemical and spectroscopic methods [10,13]. Characterization of the PCL produced the following results: mp: 59–61.5 °C. IR (KBr, cm^−1^): 2945, 2864, 1726, 1294, 1244, and 1190. ^1^H NMR (300 MHz, CDCl_3_): δ (ppm) 4.04 (t, 2H), 3.64 (t), 2.29 (t, 2H), 1.63 (m, 4H), 1.36 (m, 2H).

## 3. Results and Discussion

This section, divided into subheadings, should provide a concise and precise description of the experimental results, their interpretation as well as the experimental conclusions that can be drawn.

### 3.1. Synthesis and Characterization of Complexes **1**–**6**

Complexes **1**–**6** were obtained with high yields using a modified methodology that was reported previously [17,28,29]. The reaction between the Cu(II) and Zn(II) acetates and benzoic acid was conducted in a polar solvent (methanol) to ensure the solubility of the reactants and to obtain the respective copper and zinc benzoates via elimination of acetic acid. Once this first step was completed, the ligands derived from Pz were dissolved in toluene and afterwards added dropwise to the copper and zinc benzoate solution.

The complexes were isolated by precipitation. The methanol was removed and replaced with cold pentane to induce the formation of aggregates from the bulk of the solutions that corresponded to the desired products. 

### 3.2. Mass Spectrometry and Elemental Analysis

The results of the mass spectrometry analysis of the complexes in all cases exhibited signals that were assigned to ionization adducts of the **Pz**-derived ligands and were the most intense peaks from the chromatographic profiles. Nevertheless, the molecular ion peak was not observed in any case. Table 1 summarizes the assignments for the most abundant signals that were observed and the mass spectra that were obtained for all of the complexes (Figures S31–S36, Supplementary Materials). 

From the elemental analysis of complexes **1**–**6**, the authors determined that the stoichiometric ratio between the ligands and the metallic center was 2:1 in all cases and that the obtained percentages of nitrogen, hydrogen, and carbon in the samples were in concordance with the proposed molecular formulas.

### 3.3. Infrared and NMR Spectroscopy

The vibrational bands of the ligands were shifted or disappeared due to coordination with the metal center (Figures S6–S11, Supplementary Materials). Specifically, in all cases the carbonyl vibration (C=O) from benzoic acid was shifted to lower wavenumbers compared with the IR spectra of the free ligand (1705 cm^−1^) [30]; the band assigned to the stretching O–H bond in the benzoic acid was not observed due to the formation of metal carboxylates [28]. An increase was observed in the intensity of the bands corresponding to C=C stretching in aromatic systems (1400–1600 cm^−1^) that were due to the overlap of the signals from pyrazolic and benzoic fragments in the complexes [31].

**Figure 1:**
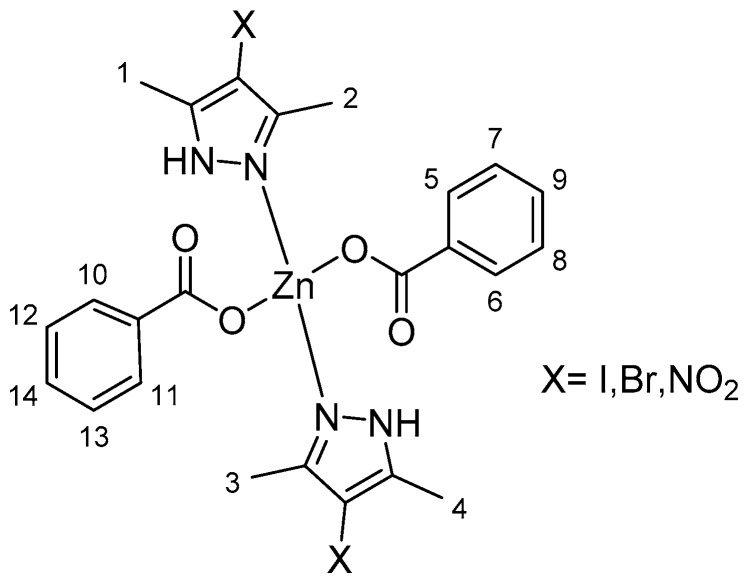
Numeration of protons for zinc complexes.

The ^1^H NMR spectra for complexes **1**, **3**, and **5** indicated shifts to higher fields in the signals assigned to protons of the methylene (CH_2_) groups in the pyrazole due to their coordination with zinc(II) (Figures S20–S22, Supplementary Materials). In all cases, the hydroxyl group H from the benzoic acid was not observed, thus the coordination of the AB to the metallic center was confirmed (Figure S15, Supplementary Materials). The integral values of the protons were in agreement with the proposed structures, as shown in Table 2 and the numeration used in Figure 1.

Alternatively, characteristic shifting of the signals assigned to the carbon atom in position 4 of the pyrazolic fragments due to the insertion of Br^−^, I^−^, and NO_2_^−^ groups was observed in the ^13^C NMR spectra (Figures S23–S25, Supplementary Materials). Specifically, shifts to higher fields for **1** and **3** (92.85 and 62.59 ppm, respectively) were observed compared with the chemical shift for carbon 4 in free **Pz** (103.94 ppm) [31]; this behavior can be explained by the heavy atom effect [32,33]. In contrast, the signal for this carbon in **5** shifted to a lower field (131.2 ppm) due to the strong electron withdrawing nature of the NO_2_^−^ group in the heterocyclic ligand. 

### 3.4. Thermal Analysis

**Figure 2:**
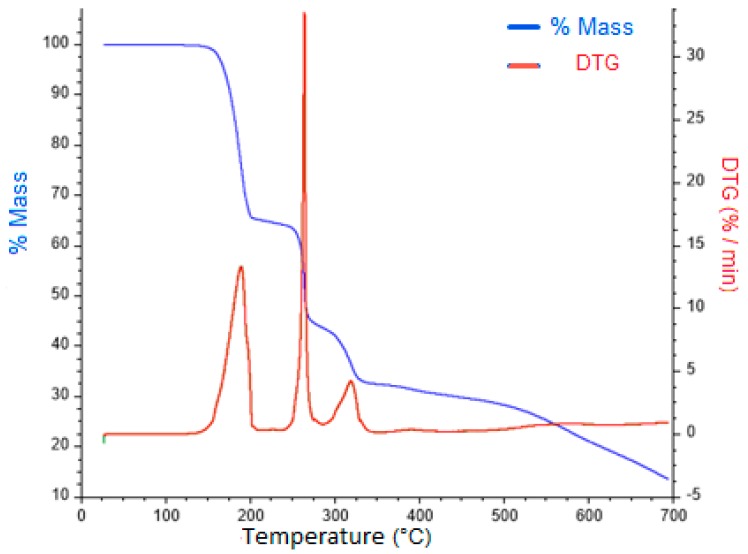
TGA and DTG for complex **4**.

A loss of mass corresponding to pyrazole fragments was observed for all of the complexes over a temperature range from 160–250 °C that was followed by the elimination of benzoic acid at higher temperatures (Figures S26–S30, Supplementary Materials). The stages of decomposition as the decomposition products formed and the weight loss percentages are proposed, because the detection of these fragments was not possible. Figure 2 shows the TGA curve for complex **4** as a representative example. 

A weight mass reduction of 29.81% was observed at 194 °C, which implies a loss of an equivalent of **I-Pz** (29.64% calculated). In a second stage, at approximately 265 °C, a probable loss of the second **I-Pz** ligand was observed as the mass percentage of **4** was reduced to 40.70% versus 40.72% (calculated).

The proposed thermal decomposition patterns for all complexes are based on the dative nature of the coordination bond between the N-donor ligands and the metal center. Since the interactions (N-donor ligand to the metal) are weaker than the covalent bond between the benzoate and the metallic center [34], fragments from the **Pz** derivatives are lost at lower temperatures than the benzoate fragments.

### 3.5. Single-Crystal X-ray Analysis

**Figure 3:**
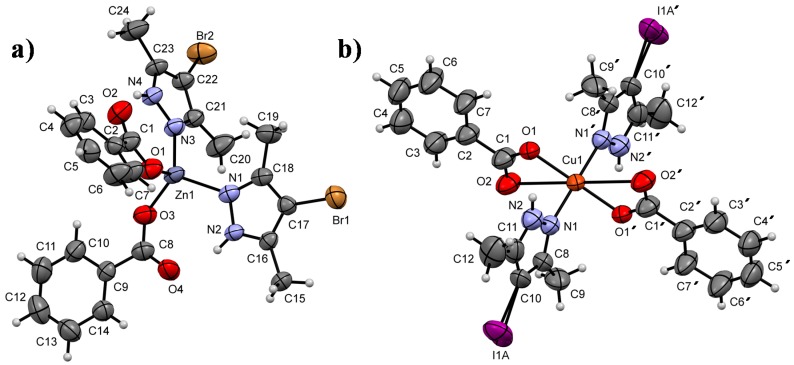
ORTEP plots of compounds (**a**) **1** and (**b**) **4**, showing displacement ellipsoids drawn at the 50% probability level. H atoms are shown as small gray spheres of arbitrary radii. Selected bond lengths (Å): (**1**): Zn1–O1 1.935(4), Zn1–O3 1.939(4), Zn1–N1 2.027(5), and Zn1–N3 2.001(5); (**4**): Cu1–O1 1.944(4), Cu1–O2 2.760(4), and Cu1–N1 1.989(5). Selected bond angles (°): (**1**): O1–Zn1–O3 106.66(17), O1–Zn1–N1 104.46(19), O1–Zn1–N3 115.60(18), O3–Zn1–N1 110.34(15), O3–Zn1–N3 108.15(18), and N1–Zn1–N3 111.5(2); (**4**): O1–Cu1–O2 52.02(15), O1–Cu1–N1 89.82(17), O1–Cu1–O1′ 180.00, O1–Cu1–O2′ 127.98(15), O1–Cu1–N1′ 90.18(17), O2–Cu1–N1 83.20(18), and O2–Cu1–N1′ 96.80(18).

Single crystals of **1** and **4** were obtained by slow diffusion of a saturated diethyl ether solution over a separated portion of tetrahydrofuran in a confined crystallization chamber. The crystallographic data for complexes **1** and **4** are summarized in Table 3. The molecular structures of both complexes are considerably different, despite the similarity of the ligands. A benzoate group is common to both compounds, and the pyrazole derivatives differ only by the halogen substituents, Br and I in compounds 1 and 4, respectively. In structure 1, the central Zn atom is coordinated to two equivalent Br-pyrazole units and two benzoate moieties. This four-coordination geometry describes a tetrahedral polyhedron [35,36] with an average volume of 1331.3(4) Å^3^ and a mean tetrahedral quadratic elongation of λ = 1.005 (Figure 3a). In the case of compound 4, the structure is characterized by a six-coordination geometry that is exhibited by the metal. The central Cu-cation is coordinated to two benzoate groups and two I-pyrazole units forming an octahedral configuration with an average volume of 5489.1(11) Å^3^ and a mean octahedral quadratic elongation of λ = 1.246 (Figure 3b). From a general point of view, the central metallic atoms in both molecules are coordinated to the same number of ligands. However, the benzoate fragment shows monodentate and bidentate behaviors in compounds **1** and **4**, respectively. This difference could be a result of the conformational distribution of the cis and trans ligands for structures **1** and **4**, respectively (see Figure 3). In the case of compound 4, the crystal has a Z′ value of 0.5, which is due to the coincident symmetry of the molecule with an inversion center that is present at the center of the molecule at (1/2, 1/2, 0). This point of symmetry relates the pyrazole and benzoate ligands in the *trans* conformation. These structural characteristics influence not only the molecular conformation of **1** and **4** but also their supramolecular arrangements. The intramolecular hydrogen bonds N2–H2···O4 and N4‒H4A···O2 have a strong interaction in the molecular structure of 1 with short H···O distances (1.85–1.86 Å), whereas the intramolecular C9‒H9A···O1^i^ [symmetry code: (i) 1−x,1−y,−z] hydrogen bonds interact in the conformation of 4 with H···O short distances (2.39 Å), but to a lesser extent compared with compound 1.

**Figure 4:**
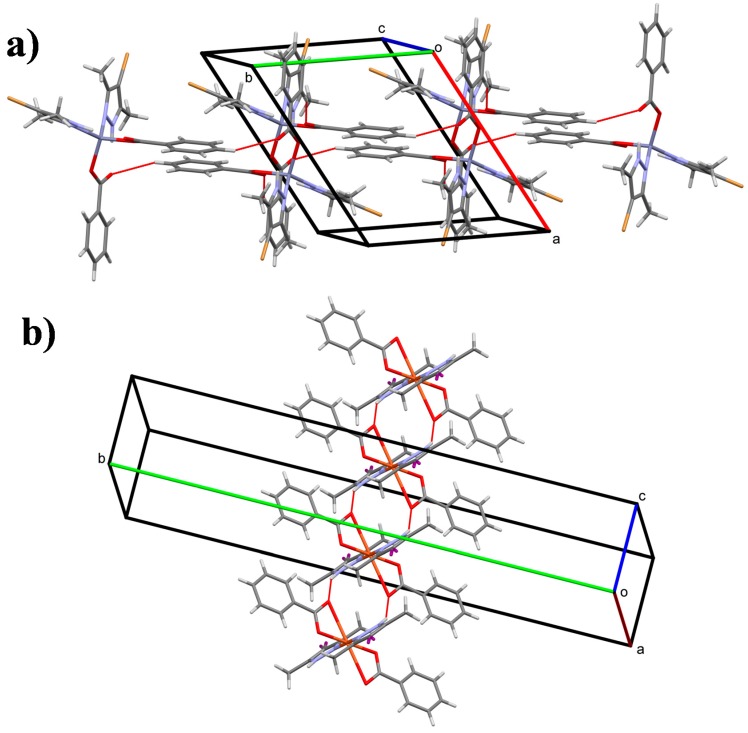
Crystal structure of (**a**) complex **1** and (**b**) complex **4** showing the formation of hydrogen-bonded chains (dashed lines) along the [010] and [001] directions, respectively.

In the crystal of **1**, the packing is directed by weak C–H···O interactions, as shown in Figure 4a. Pairs of inversion-related molecules, connected by two equivalent weak C5–H5···O4^ii^ [symmetry code: (ii) 1−x,1−y,1−z; H···O = 2.74 Å] interactions form infinite chains of molecules, that are connected further by two equivalent weak C10–H10···O2^iii^ [symmetry code: (iii) 1−x,2−y,1−z; H···O = 2.81 Å] interactions along the [010] direction. Weak dipolar and van der Waals forces between neighboring chains contribute to the formation of the three-dimensional architecture.

The crystal structure of compound **4** is controlled by strong N2–H2···O2^iv^ [symmetry code: (iv) x,y,−1+z; H···O = 2.00 Å] hydrogen bonds that join molecules in chains running along the [001] direction (Figure 4b). This feature defines a marked difference between the forces that keep the molecules assembled (i.e., weak and very strong interactions in **1** and **4**, respectively). In the [100] and [001] directions, C12‒H12A···Cg_1_^v^ [symmetry code: (v) 5/4−y,1/4+x,1/4−z; H···Cg_1_= 2.95 Å (Cg_1_ is the centroid of the C2/C7 ring)] contacts connect neighboring chains to form the three-dimensional crystal structure.

### 3.6. Polymerization of ε-Caprolactone

Complexes **1**–**5** exhibited high activity for ROP of CL, and they produced linear polycaprolactones with high crystallinity, indicating excellent control over the polymerization reaction and, therefore, low occurrence of transesterification side reactions [2]. The yields for the reactions and calorimetric and thermal characterizations of the materials are shown in Table 4. 

All polymers showed melting points characteristic of PCL (Figures S47–S51, Supplementary Materials). As a general result, initiators **1**–**5** produced a significant improvement in terms of the ratio of monomer:initiator (100:1) compared to related Cu(II) and Zn(II) complexes [17]. This result is likely due to the insertion of EWG in the ligands, which makes the metal center more electron-deficient, increasing the reactivity of the coordinated caprolactone. 

^1^H NMR analysis of the polymers helped to confirm the formation of PCL. The spectrum contains the following signals which were assigned to methylene group protons: (400 MHz, CDCl_3_) δ: 4.06 (–OCH_2_–), 2.31 (–CH_2_C=O), 1.64 (–CH_2_–), and 1.38 (–CH_2_–) ppm (Figures S37–S41, Supplementary Materials). The signal observed at 3.65 ppm corresponds to a hydroxymethylene chain-ending group, which confirms the formation of a linear chain polyester [7].

**Figure 5:**
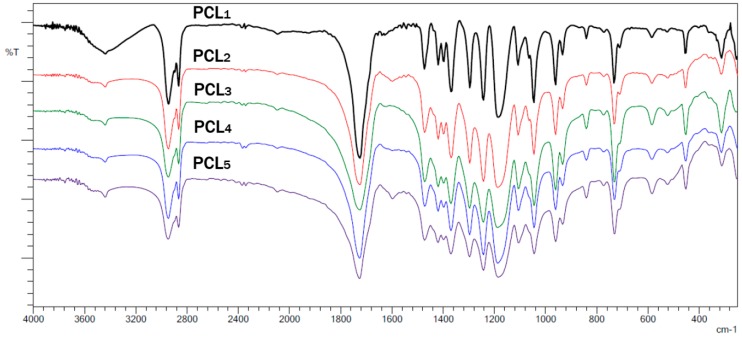
FTIR spectra of the PCLs obtained using **1**–**5**.

FTIR spectra of the obtained polymers exhibited absorption bands that correspond to a linear aliphatic polyester. Figure 5 shows the FTIR spectra of the PCLs obtained.

Figure 5 shows two intense bands at 2941 and 2868 cm^−1^ that correspond to the stretching of C–H bonds of methylene carbons. There is also an intense signal at 1728 cm^−1^ that is due to absorption by the carbonyl group in the PCL, and stretching bands at 1290 and 1241 cm^−1^ from the C–O bond of the ester group [37]. 

Unfortunately, complex **6** did not produce any activity for the ROP of CL under the proposed conditions. This result is due to the fact that no active species were formed using this initiator. The zinc complexes produced higher yields than their copper analogues (see Table 4); this behavior can be attributed to the Cu(II) species being less electropositive than Zn(II) [38]. 

The curves obtained from the gel permeation chromatography (GPC) analysis of polycaprolactones produced using **1**–**4** exhibited a bimodal distribution which could suggest a non-unique pathway of initiation [39], whereas the GPC analysis of the polymer obtained with **5** as initiator produced a monomodal distribution. The polydispersity index values for the materials obtained were lower (Table 5) compared with PCLs obtained using previously reported zinc- and copper-related complexes [11,17]. In contrast, it has been reported that the polydispersity is much better controlled at lower temperatures [40]. Nevertheless, the reactions were performed at 110 °C, obtaining low polydisperse PCL.

The average molecular weight (M_n_) for all of the PCLs was between 17,932 and 32,688. Therefore, they could be classified as low molecular weight polymers [40], which could also suggest that the chain termination step with initiators **1**–**5** is effective.

The GPC analysis of the polymer obtained with **5** as initiator produced a monomodal distribution (Figure S60, Supplementary Materials). The high crystallinity of the obtained polymers along with their low molecular weight confers them potential applicability in the fabrication of biodegradable thin and ultra-thin films. This is due to the improvement of the barrier and permeation properties of polymeric films when their percentage of crystallinity has higher values and their average molecular weight decreases [41]. Therefore, these polymers could be applied or implemented in the design of biodegradable packaging [42]. In general, the PCLs obtained showed a lower dispersion, a higher molecular weight and high degrees of crystallinity compared with other polycaprolactones obtained using previously reported metal catalysts by the authors’ research group and PCLs that are commercially available [9,10,13,16,42].

## 4. Conclusions

In summary, the authors synthesized and characterized new metal complexes that were derived from 3,5-dimethylpyrazole and benzoic acid ligands with the insertion of strong electron withdrawing substituents on the N-donor ligands. The structures of **1** and **4** were determined by single-crystal X-ray diffraction and exhibited tetrahedral and octahedral geometries, respectively. Complexes **1**–**5** were active in the ring-opening polymerization (ROP) of ε-caprolactone (CL), and produced polymers that were mainly linear with narrow molecular weight distributions, high crystallinity and decomposition temperatures above 260 °C. In general, the presence of bromide, iodide, and nitro groups at the fourth position of the pyrazole ring produced an improvement in terms of the ratio of monomer:initiator compared to Cu(II) and Zn(II) complexes that have been reported to date. Finally, no information was provided regarding the effect of the substituents on the textural properties of the PCLs obtained.

## Figures and Tables

**Table 1:** Most populated peaks and their assignments from the mass spectrometry analysis of complexes **1**–**6**.

Complex	Found, *m*/*z*	Assignment	Calculated, *m*/*z*
**1**	174.9893	[C_5_H_7_BrN_2_ + H^+^]^+^	174.9865
**2**	174.9874	[C_5_H_7_BrN_2_ + H^+^]^+^	174.9865
**3**	222.9815	[C_5_H_7_IN_2_ + H^+^]^+^	222.9727
**4**	222.9857	[C_5_H_7_IN_2_ + H^+^]^+^	222.9727
**5**	142.1591	[C_5_H_7_N_3_O_2_ + H^+^]^+^	142.0611
**6**	142.0618	[C_5_H_7_N_3_O_2_ + H^+^]^+^	142.0611

**Table 2:** Chemical shifts in ^1^H NMR spectra of the hydrogen atoms in complexes **1**, **3**, and **5**, (*n*) = Integral values (See Figure 1).

Complex	δH_1-4_	δH_7,8,12,13_	δH_9,14_	δH_5,6,10,11_
**1**	2.28 (11.65)	7.40 (4.01)	7.48 (2.02)	8.12 (4.02)
**3**	2.12 (11.66)	7.45 (4.18)	7.51 (1.90)	7.98 (3.96)
**5**	2.12 (11.67)	7.43 (3.91)	7.51 (2.09)	7.96 (4.07)

**Table 3:** Crystallographic data and experimental details for complexes **1** and **4**.

Complex	1	4
Chemical Formula	C_24_H_24_Br_2_N_4_O_4_Zn	C_24_H_24_I_2_N_4_O_4_Cu
M*_r_*	657.66	749.82
Crystalline System	Triclinic	Tetragonal
Spatial Group	*P-*1	*I*4_1_/*a*
Temperature (K)	298(2)	298(2)
*a*, *b*, *c* (Å)/*a*, *c* (Å)	11.5022(19), 11.706(2), 12.459(2)	31.5581(18), 5.5116(9)
*α*, *β*, *γ* (°)	111.470(15), 101.888(14), 111.795(16)	90, 90, 90
V (Å3)	1331.3(4)	5489.1(11)
Z	2	8
Type of Radiation	MoKα	MoKα
*µ* (mm^−1^)	3.96	3.08
Crystal Size (mm)	0.38 × 0.29 × 0.21	0.21 × 0.12 × 0.09
*T*_min_, *T*_max_	0.871, 1.000	0.408, 1.000
No. of measured, independent and observed [I > 2σ(I)] reflections	153.37, 5600, 4030	29.200, 3012, 2206
*R*_int_	0.059	0.076
(sin *θ*/*λ*)_max_ (Å^−1^)	0.641	0.641
*R[F*^2^*> 2σ(F*^2^*)]*, *wR(F*^2^*)*, *S*	0.049, 0.136, 1.05	0.061, 0.193, 1.08
No. of reflections	5600	3012
No. of parameters	320	172
No. of restraints	13	69
H-atom treatment	H-atom parameters constrained	H-atom parameters constrained
Δρ_max_, Δρ_min_ (e Å^−3^)	0.77, −0.89	1.81, −0.74

**Table 4:** Yields and calorimetric and thermal characterization of the obtained PCLs.

Polymer	% Yield	Time (h) ^a^	Melting Point ^b^ (°C)	T_C_ (°C)	Crystallinity ^c^ (°C)	T_D_ (°C)
PCL_1_	99.0	26	59.9	32.8	74.4	285
PCL_2_	96.3	29	61.5	38.7	78.6	277
PCL_3_	99.6	25	60.1	33.1	72.2	261
PCL_4_	95.8	28	60.3	33.4	72.8	273
PCL_5_	92.1	28	59.9	30.6	70.2	268

^3^PCL**_(COMPLEX)_**, ^a^ Time for complete reaction with [M]/[I] =100:1 at 110 °C, ^b^ The values correspond to the first heating ramp, T_C_ = Crystallization temperature, ^c^ Calculated using a 100% crystalline PCL enthalpy of fusion equal to 136 J/g [21]. T_D_ = Decomposition temperature obtained by TGA.

**Table 5:** Molecular weight distributions and polydispersity index (PDI) of the obtained polymers.

Polymer	M_n_	M_W_ (Da)	PDI ^a^
PCL**_1_**	32,688	38,762	1.19
PCL**_2_**	31,647	37,146	1.17
PCL**_3_**	30,946	36,583	1.18
PCL**_4_**	31,770	37,763	1.19
PCL**_5_**	17,932	22,973	1.28

PCL**_(complex)_**, ^a^ PDI = M_W_/M_n_.

## Supplementary Materials

Supplementary information available at http://www.mdpi.com/2073-4360/10/11/1239/s1: characterization data of compounds, DSC and GPC curves of PCL. Crystallographic data for the structural analysis have been deposited with the Cambridge Crystallographic Data Center, CCDC-1834275 and -1834276.
