# Supplementary material for: Polymers of ε-Caprolactone Using New Copper(II) and Zinc(II) Complexes as Initiators: Synthesis, Characterization and X-Ray Crystal Structures
Source: Polymers (Basel). 2018 Nov 8;10(11):1239. doi: 10.3390/polym10111239 (PMC6401771; doi:10.3390/polym10111239)
Supplement: Supplementary file 1 [file polymers-10-01239-s001.pdf]

Supplementary Materials

Polymers of ε-Caprolactone Using New Copper(II) and Zinc(II) Complexes as Initiators. Synthesis, Characterization and X-ray Crystal Structures

Andrés F. Posada [1], Mario A. Macías [1], Santiago Movilla [1], Gian Pietro Miscione [1], León D. Pérez [2] and John J. Hurtado [1,*]

[1] Departamento de Química, Universidad de los Andes, Carrera 1 No. 18A-12, 111711, Bogotá-Colombia; af.posada@uniandes.edu.co (A.F.P); ma.maciasl@uniandes.edu.co (M.A.M); s.movilla82@uniandes.edu.co (S.M); gp.miscione57@uniandes.edu.co (G.P.M)

[2] Grupo de Macromoléculas, Departamento de Química, Universidad Nacional de Colombia, Carrera 45 No 26–85, edificio 451 of. 449, Bogotá D.C. Colombia; ldperezp@unal.edu.co

*Corresponding author.
E-mail address: jj.hurtado@uniandes.edu.co (J. Hurtado), Tel.: +57-1-339-4949 (ext. 3468)
ORCID ID: 0000-0002-0511-9719

Contents

1. Characterization of ligands and complexes

2. Characterization of polymers

1. Characterization of ligands and complexes

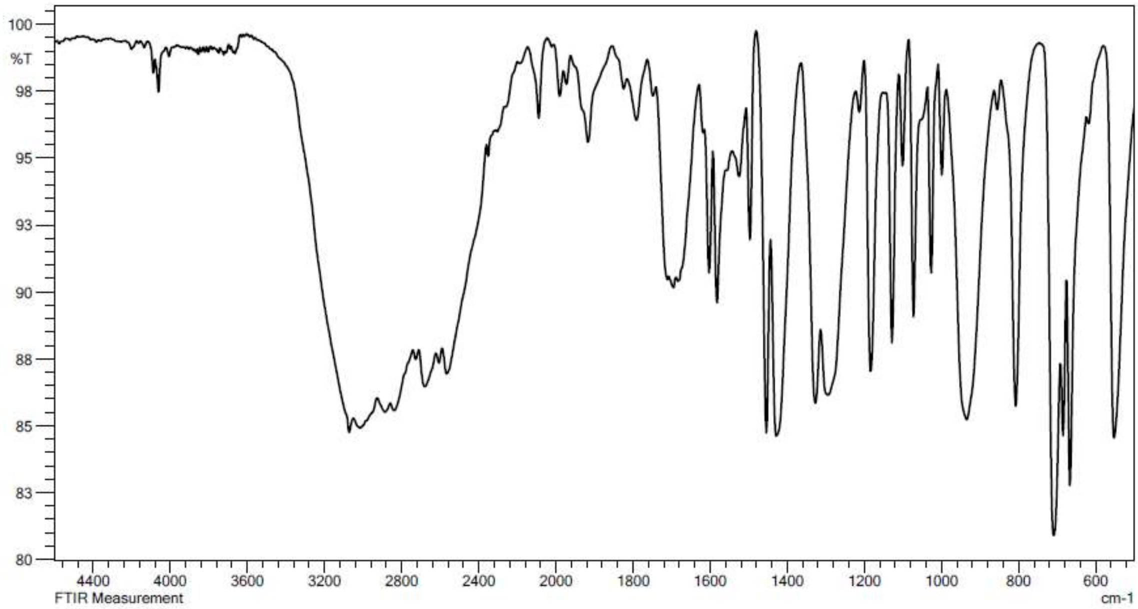

Figure S1. FT-IR (KBr) spectrum of AB ligand**.**

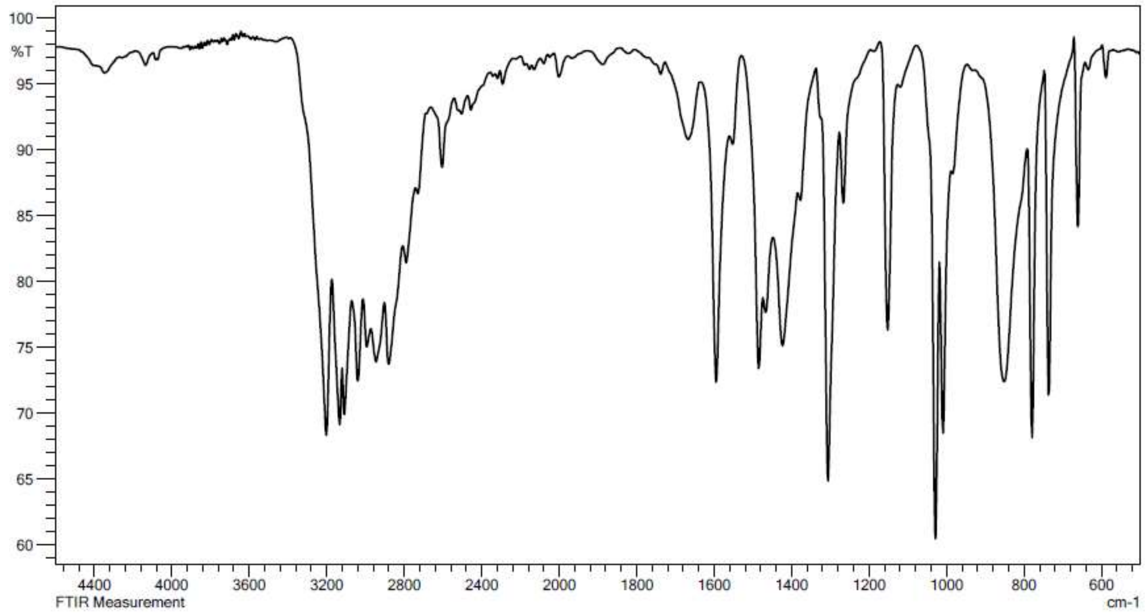

Figure S2. FT-IR (KBr) spectrum of Pz ligand**.**

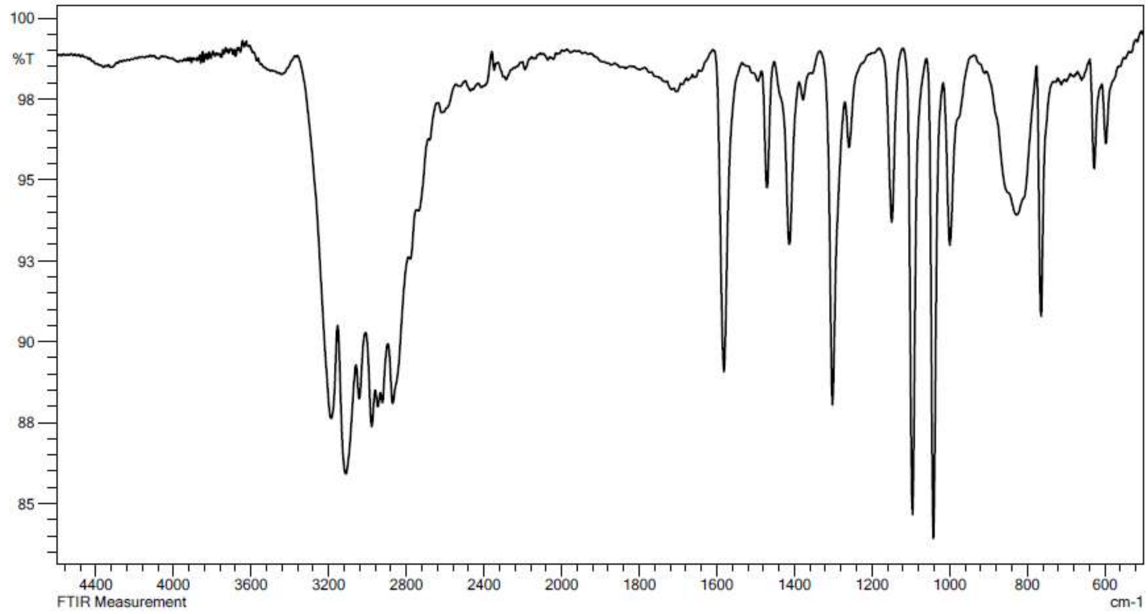

Figure S3. FT-IR (KBr) spectrum of Br-Pz ligand.

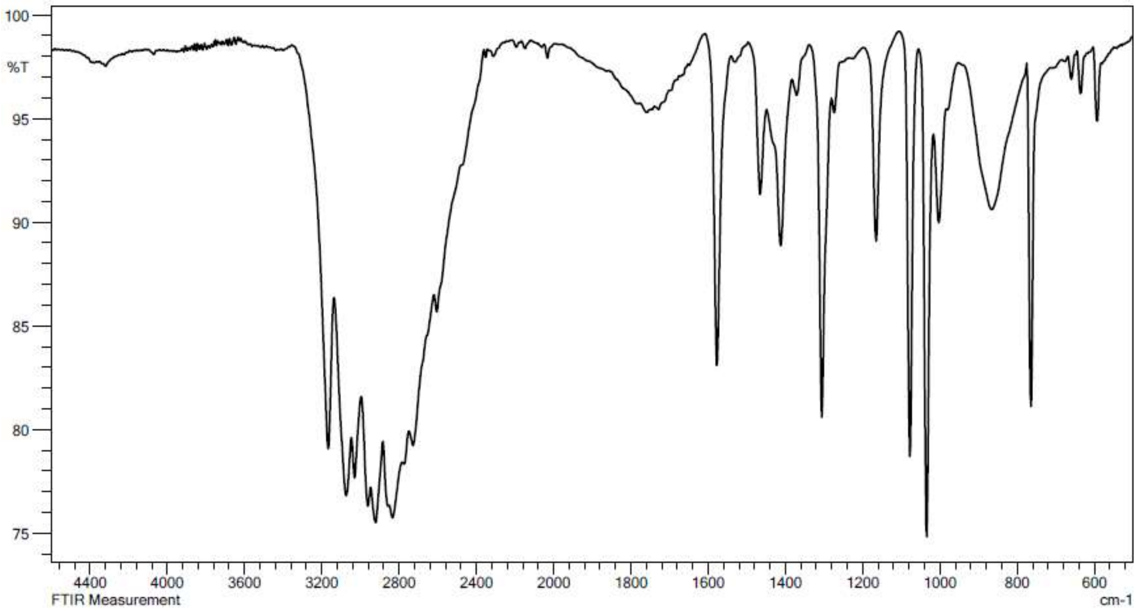

Figure S4. FT-IR (KBr) spectrum of I-Pz ligand**.**

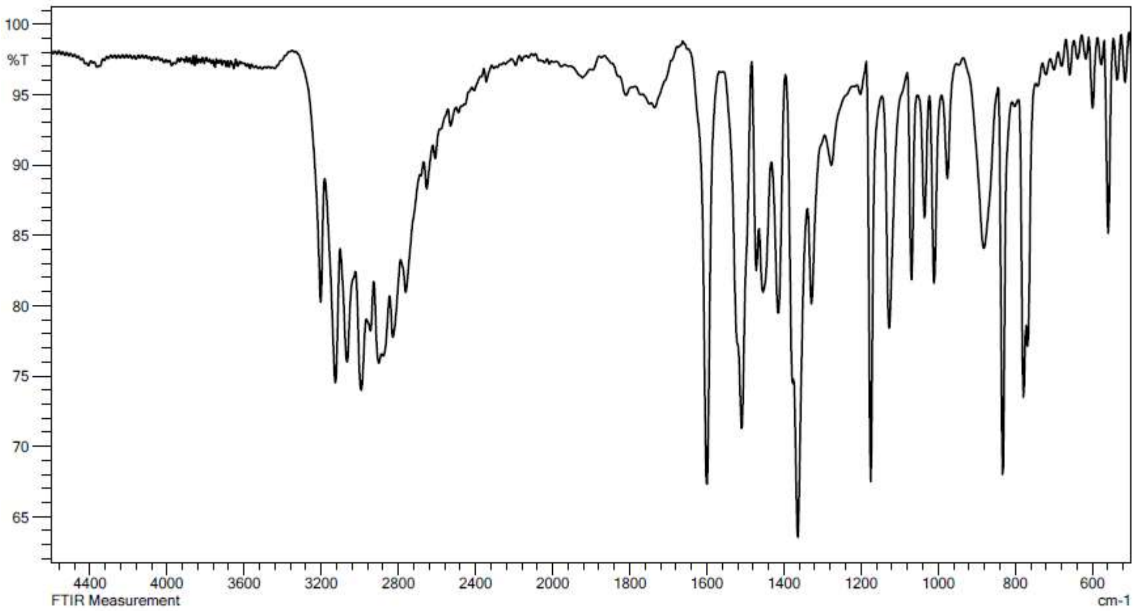

Figure S5. FT-IR (KBr) spectrum of NO_2-Pz ligand**.**

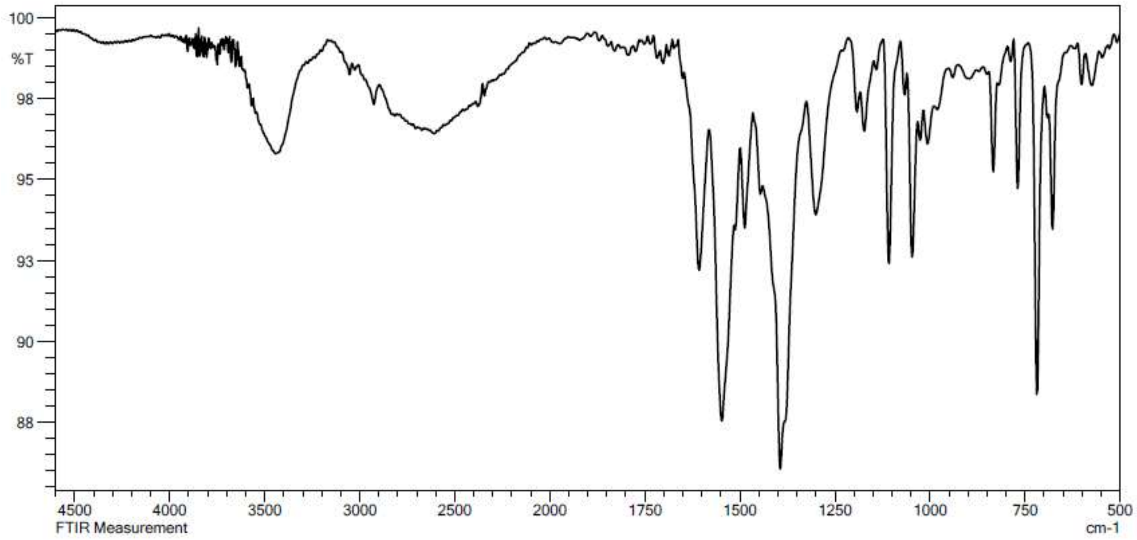

Figure S6. FT-IR (KBr) spectrum of **1.**

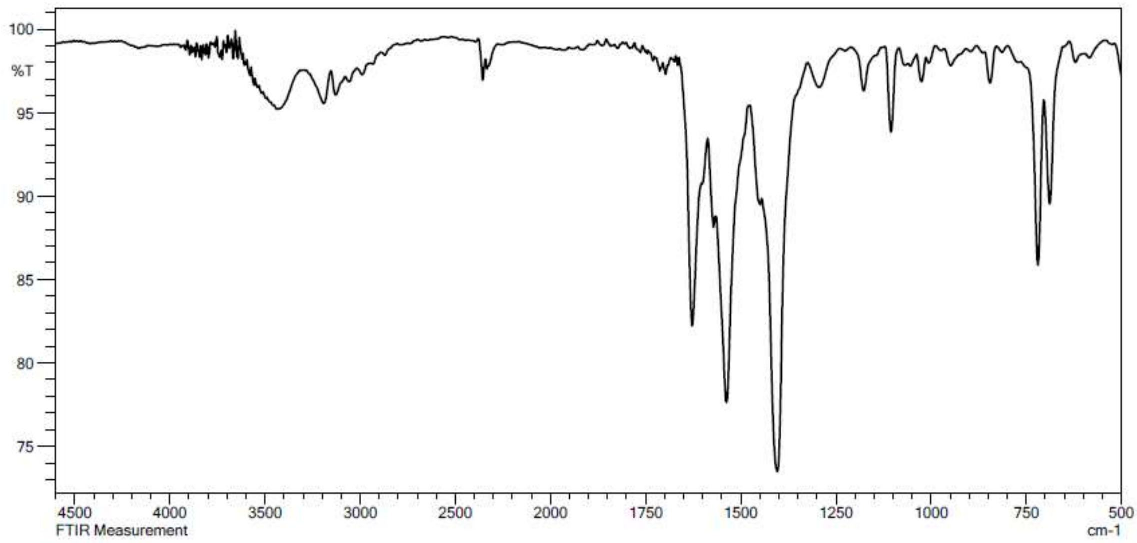

Figure S7. FT-IR (KBr) spectrum of **2.**

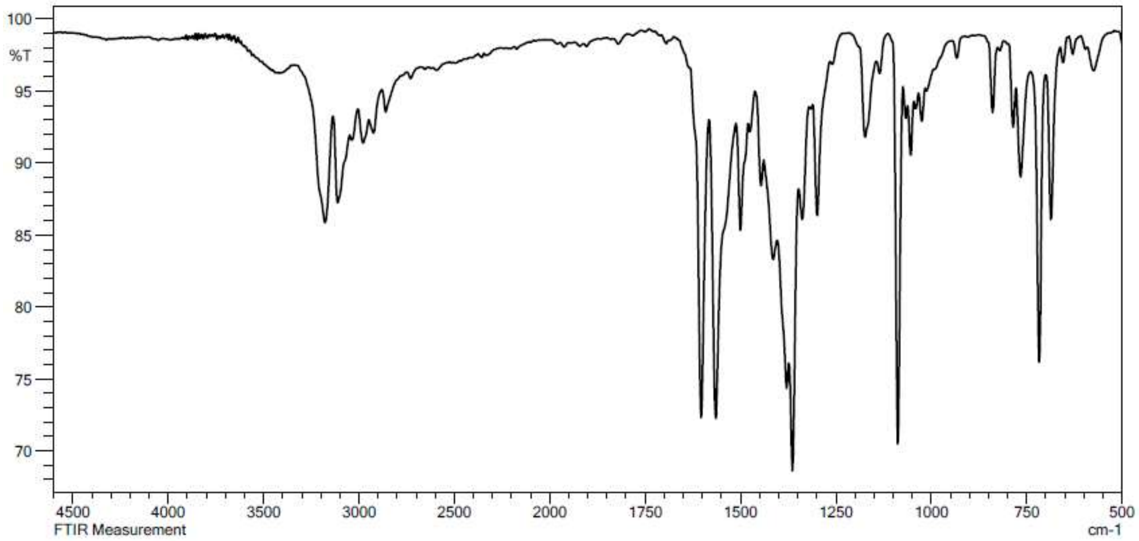

Figure S8. FT-IR (KBr) spectrum of **3.**

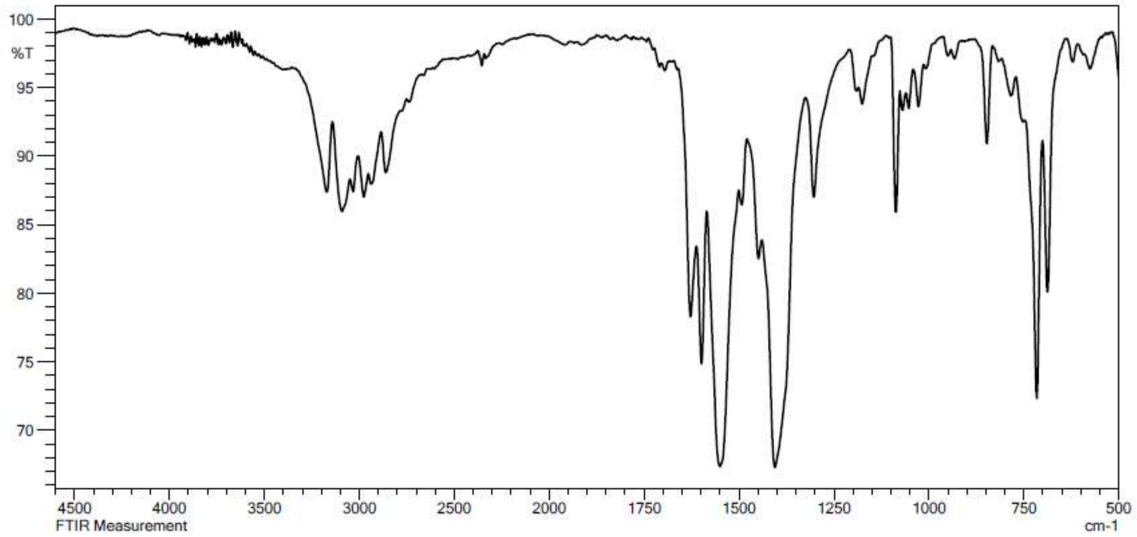

Figure S9. FT-IR (KBr) spectrum of **4.**

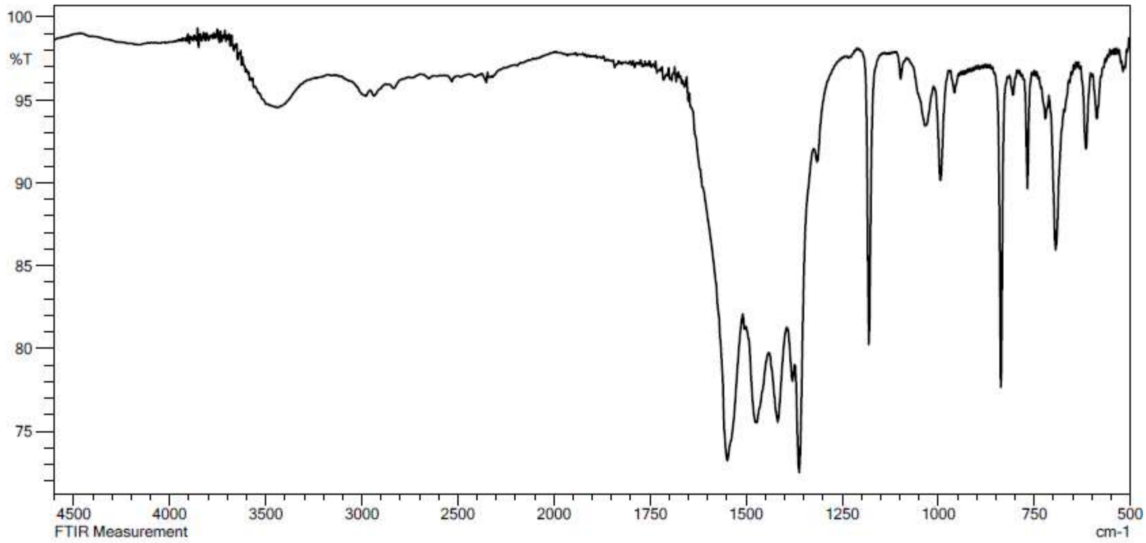

Figure S10. FT-IR (KBr) spectrum of **5.**

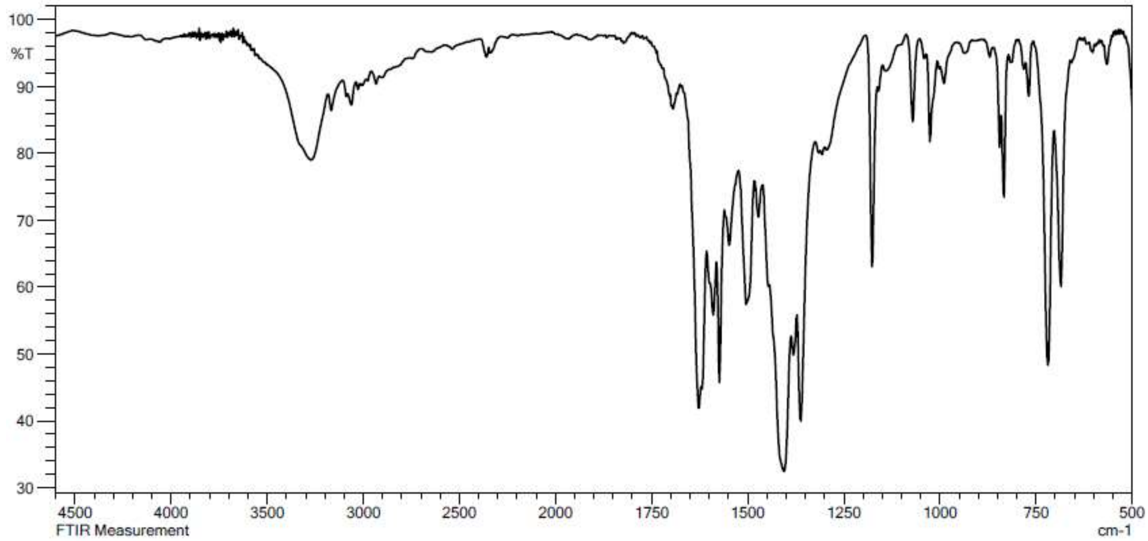

Figure S11. FT-IR (KBr) spectrum of **6.**

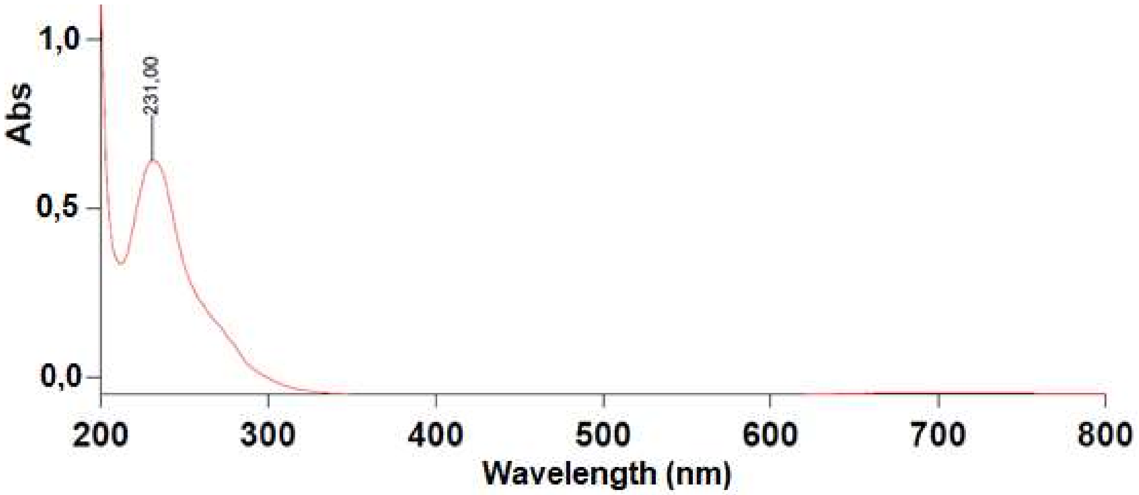

Figure S12. Absorption spectrum of **2** (2.5 x 10^{-5} M) in acetonitrile at room temperature

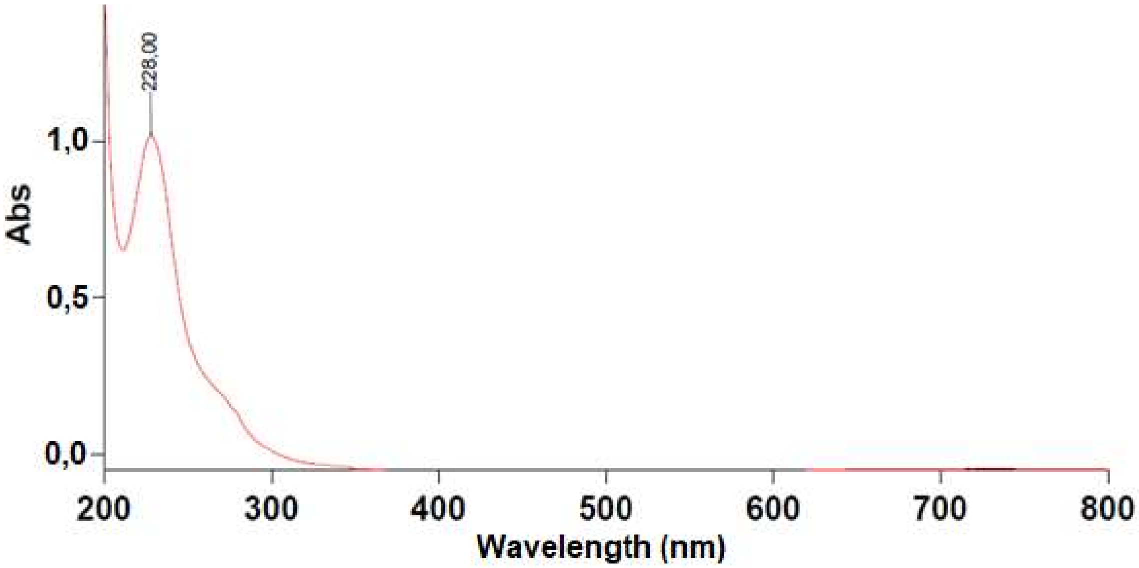

Figure S13. Absorption spectrum of **4** (5.0 x 10^{-5} M) in acetonitrile at room temperature.

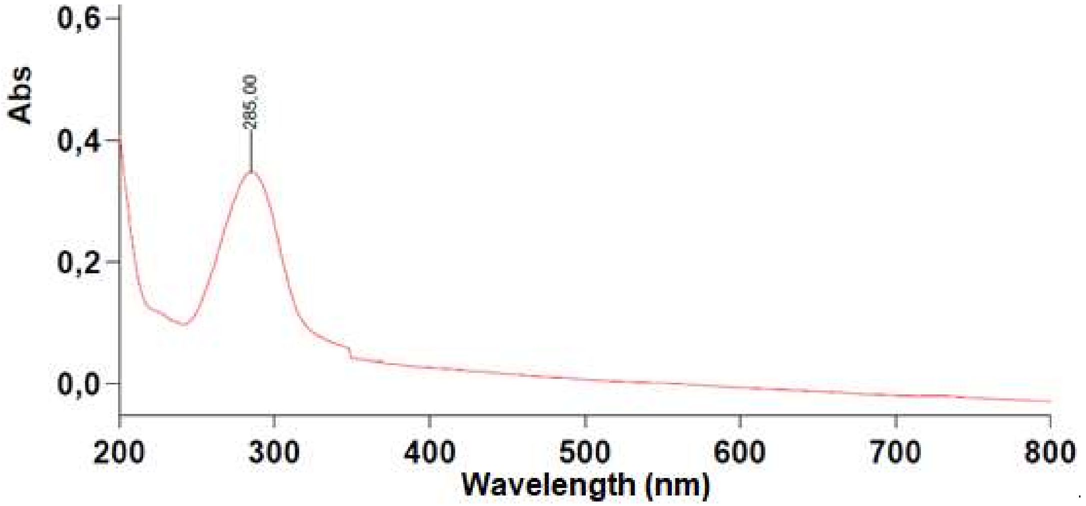

Figure S14. Absorption spectrum of **6** (2.5 x 10^{-5} M) in acetonitrile at room temperature**.**

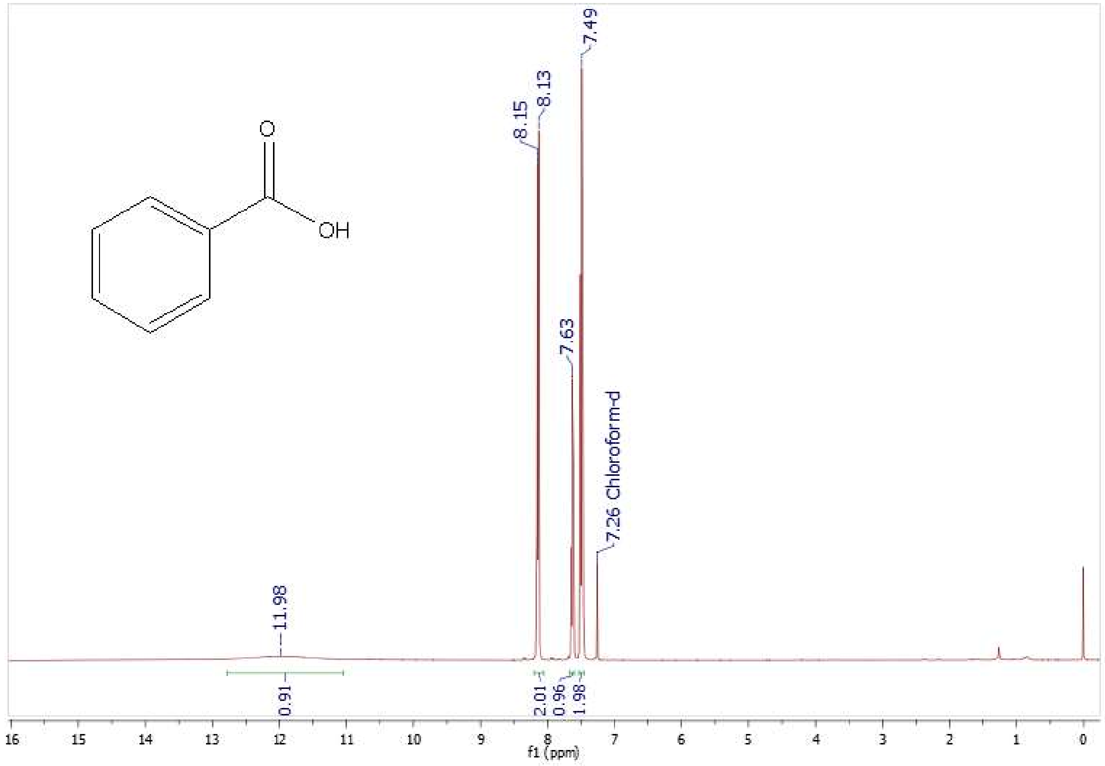

Figure S15. ^{1}H NMR spectrum of AB ligand in $CDCl_3$.

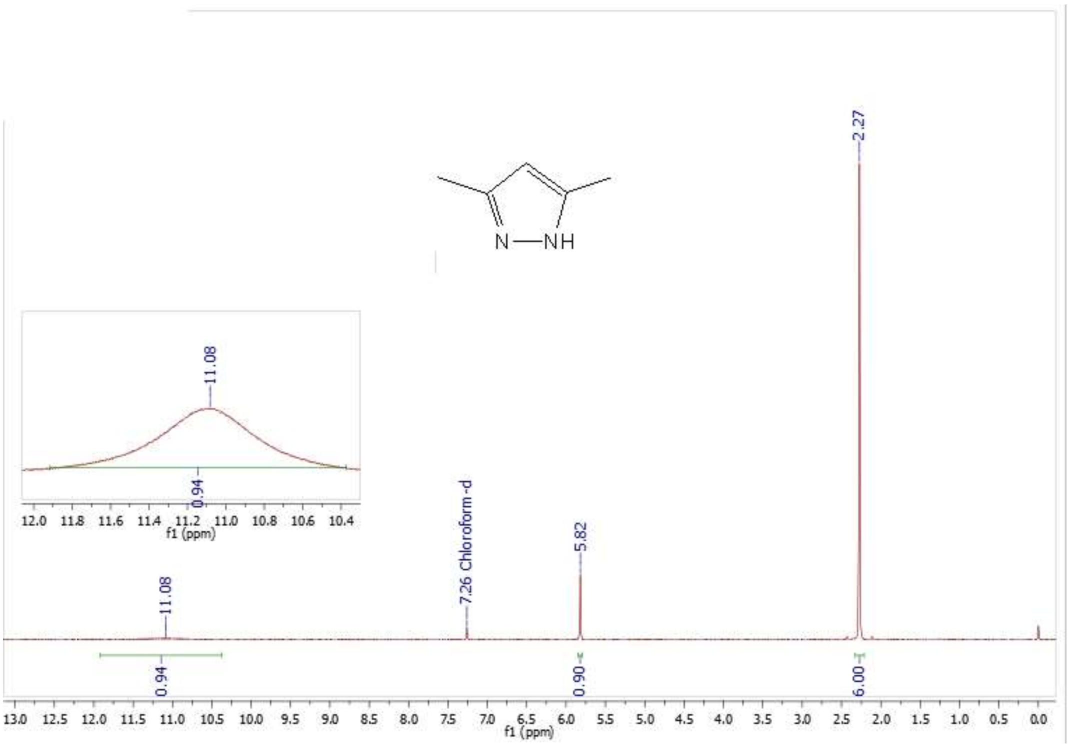

Figure S16. ^{1}H NMR spectrum of Pz ligand in $CDCl_3$**.**

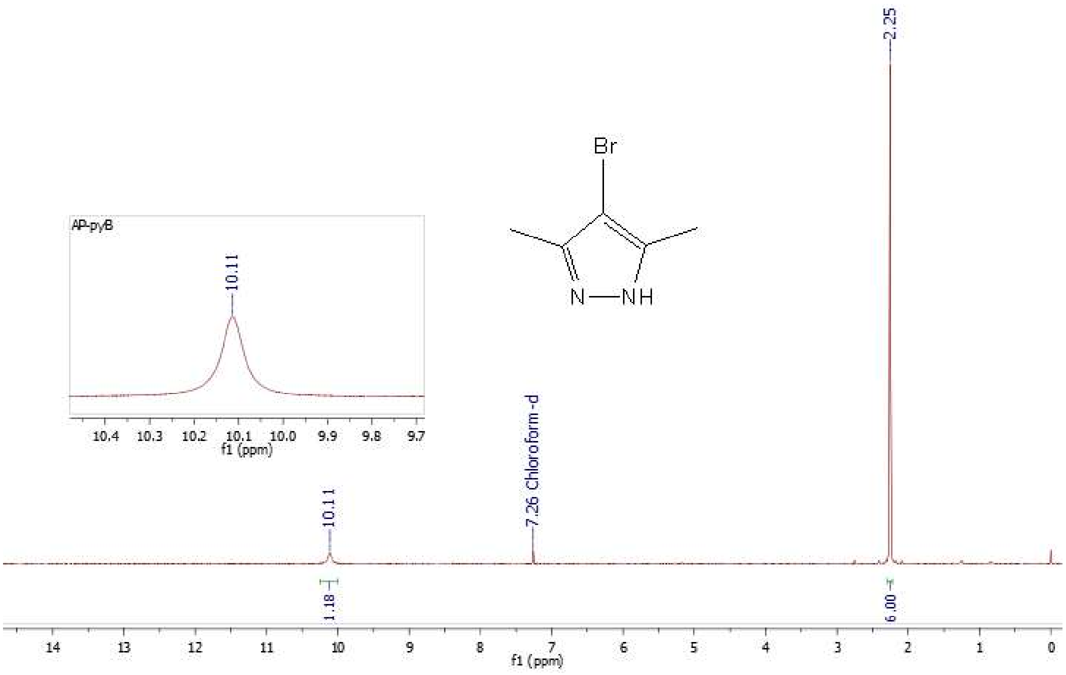

Figure S17. ^{1}H NMR spectrum of Br-Pz ligand in $CDCl_3$**.**

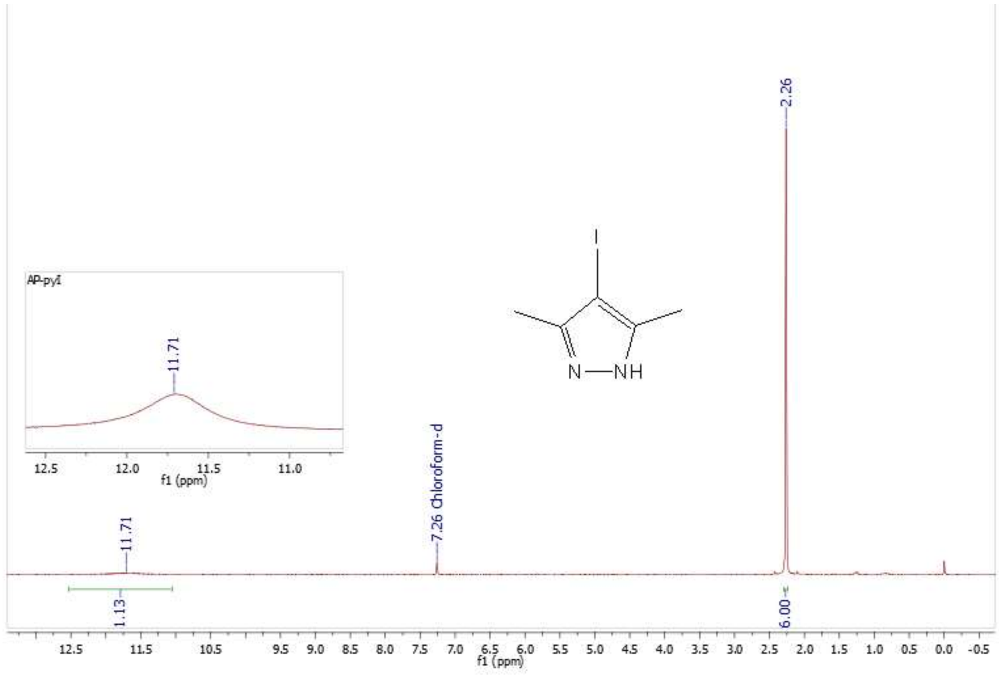

Figure S18. ^{1}H NMR spectrum of I-Pz ligand in $CDCl_3$**.**

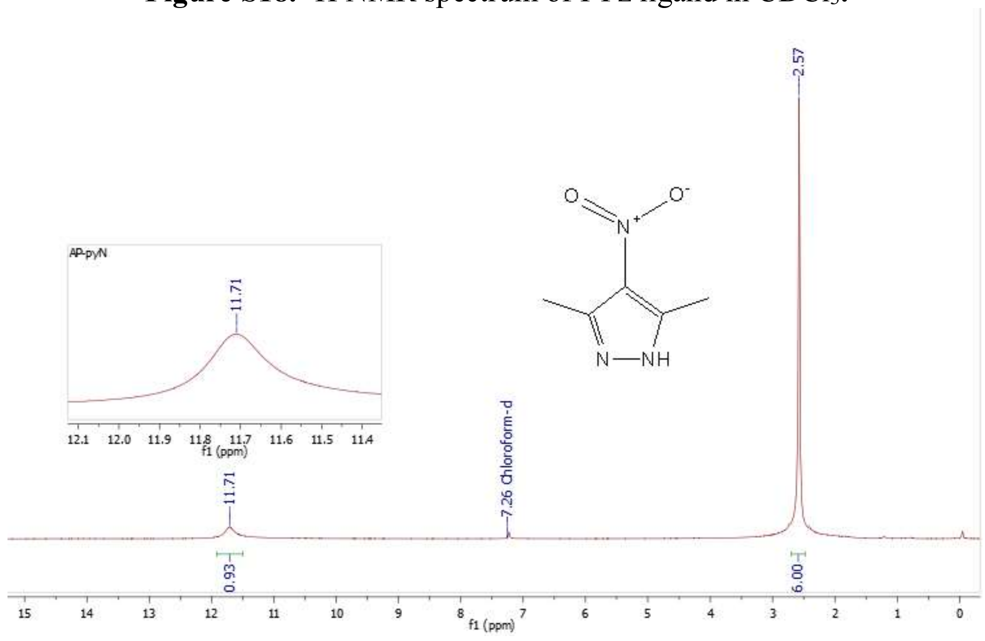

Figure S19. ^{1}H NMR spectrum of NO_2-Pz ligand in $CDCl_3$**.**

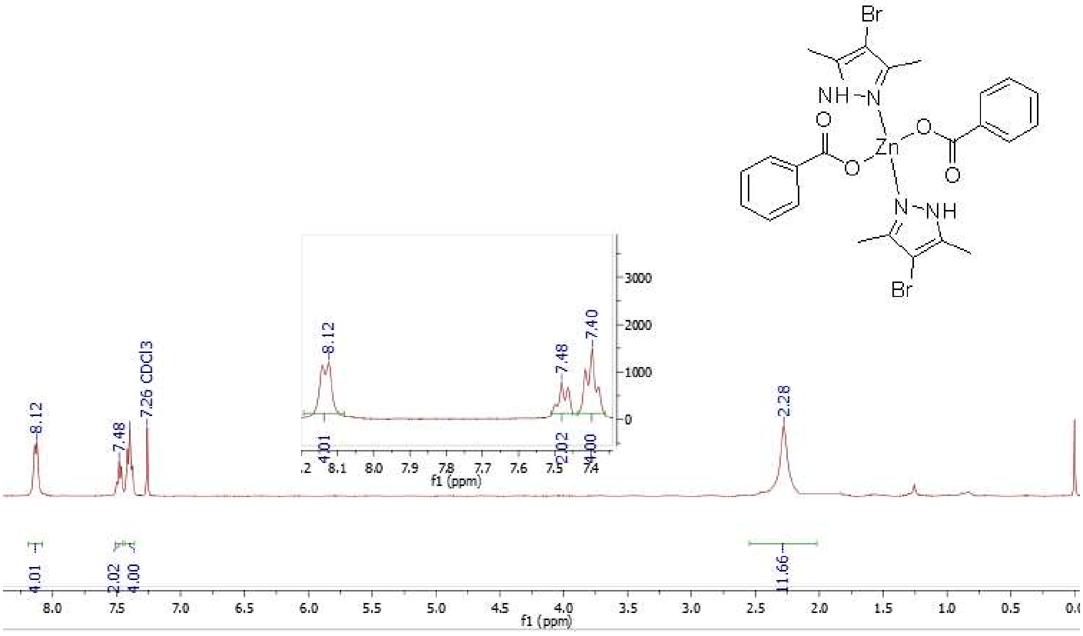

Figure S20. ^{1}H NMR spectrum of **1** in $CDCl_3$**.**

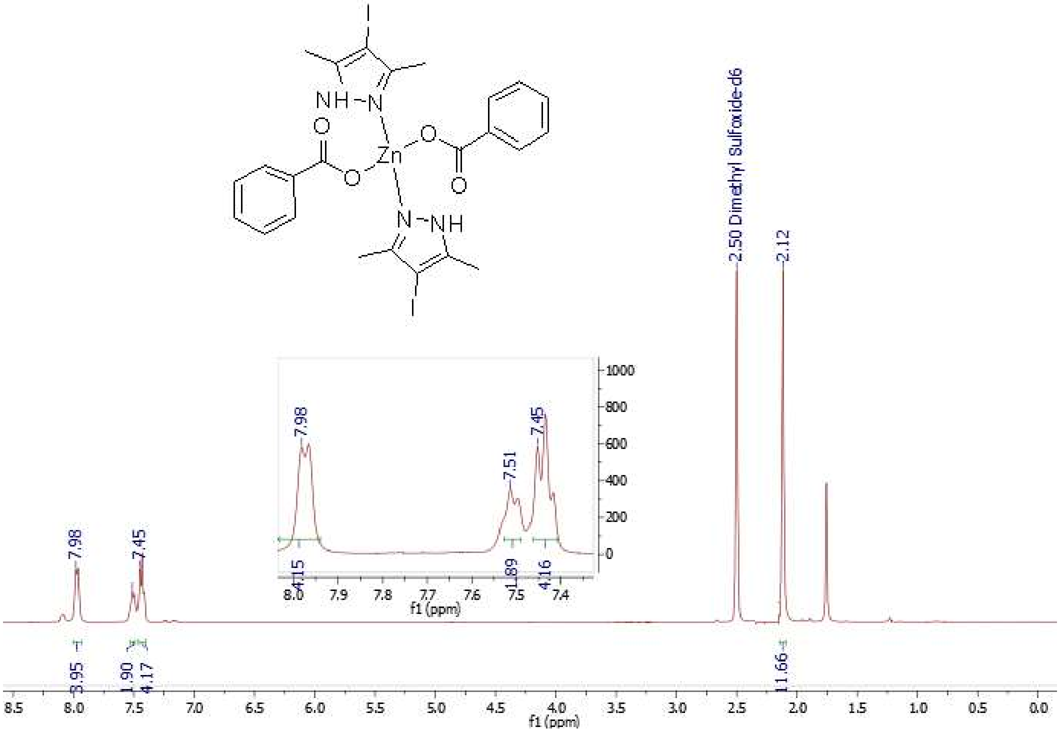

Figure S21. ^{1}H NMR spectrum of **3** in $(CD_3)_2SO$**.**

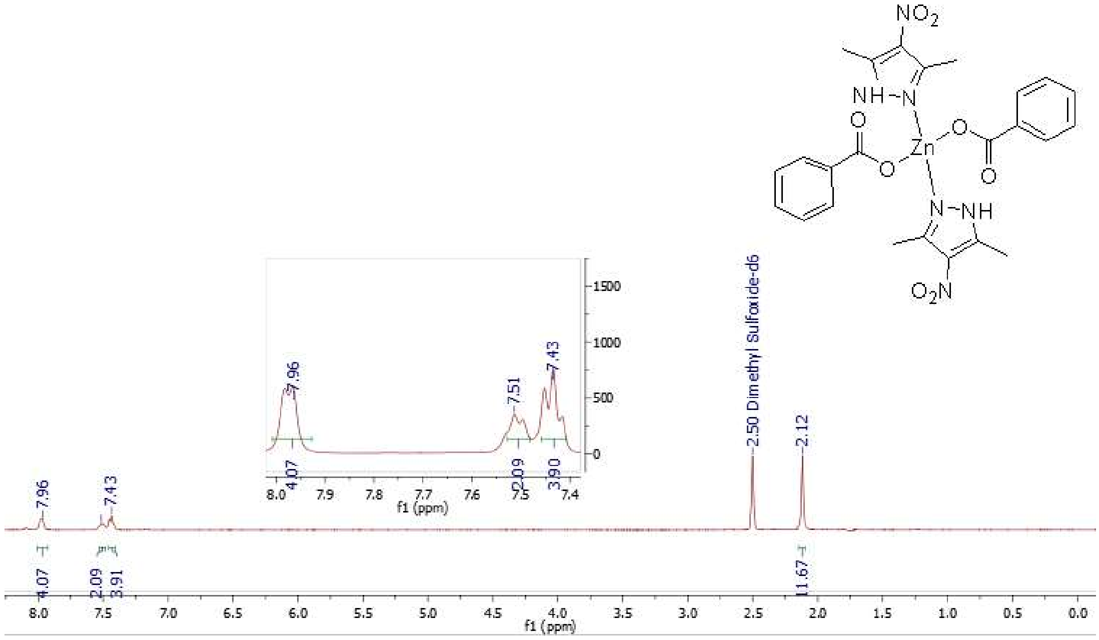

Figure S22. ^{1}H NMR spectrum of **5** in $(CD_3)_2SO$**.**

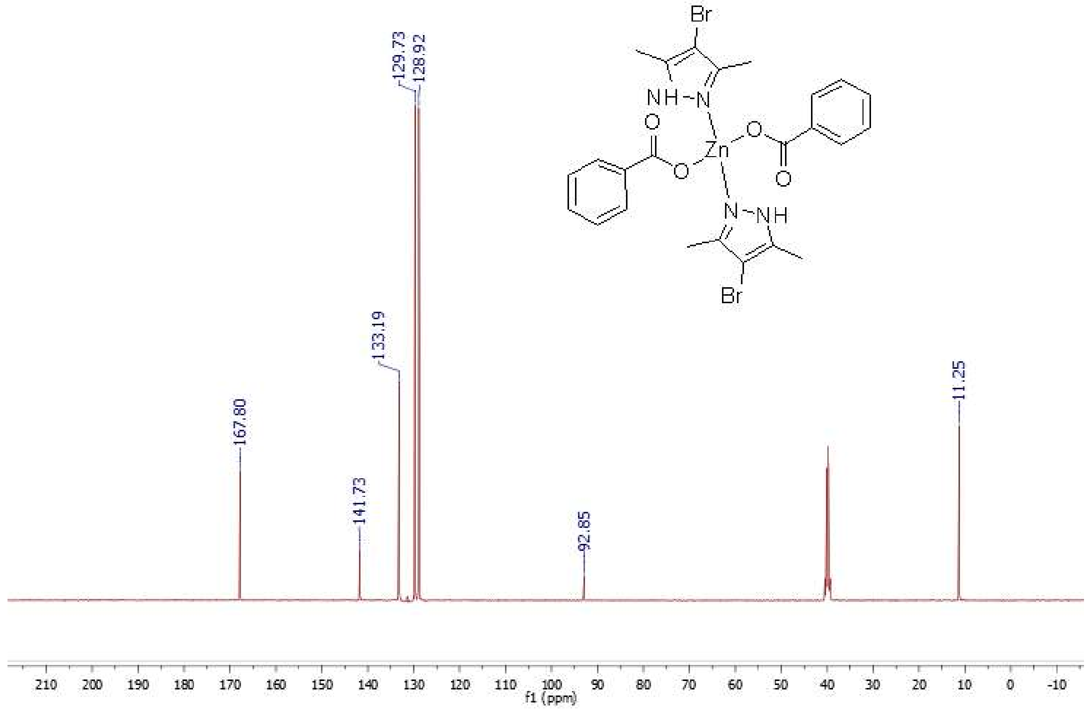

Figure S23. ^{13}C NMR spectrum of **1** in $(CD_3)_2SO$**.**

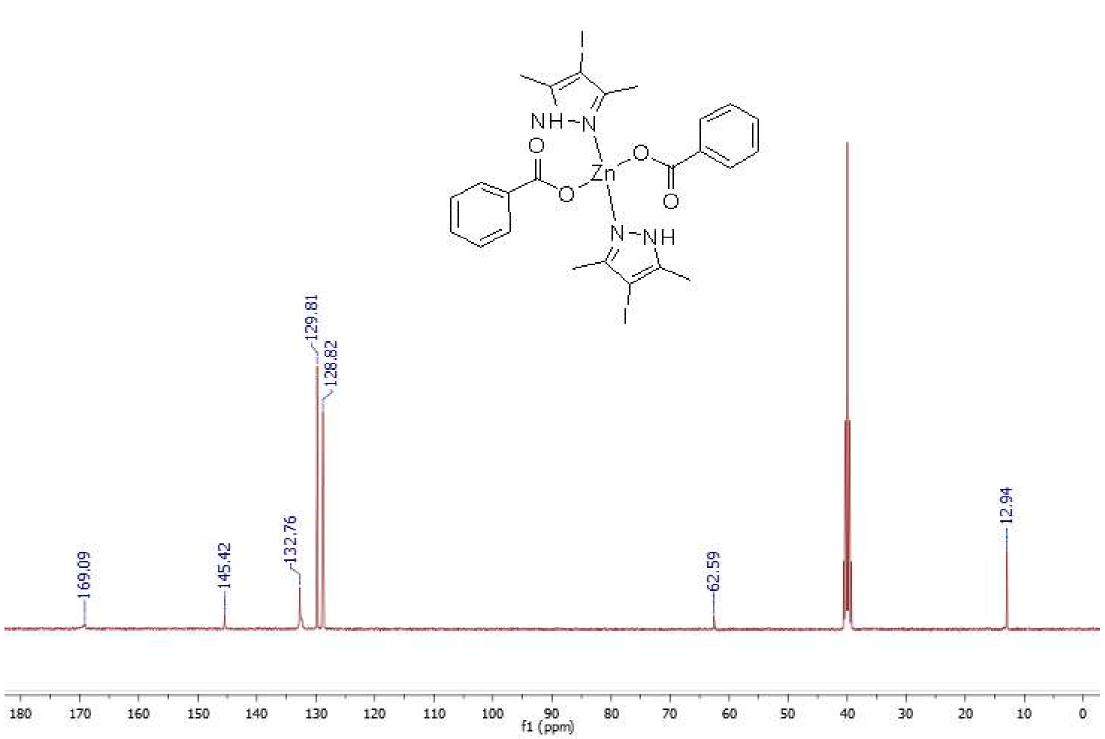

Figure S24. ^{13}C NMR spectrum of **3** in $(CD_3)_2SO$**.**

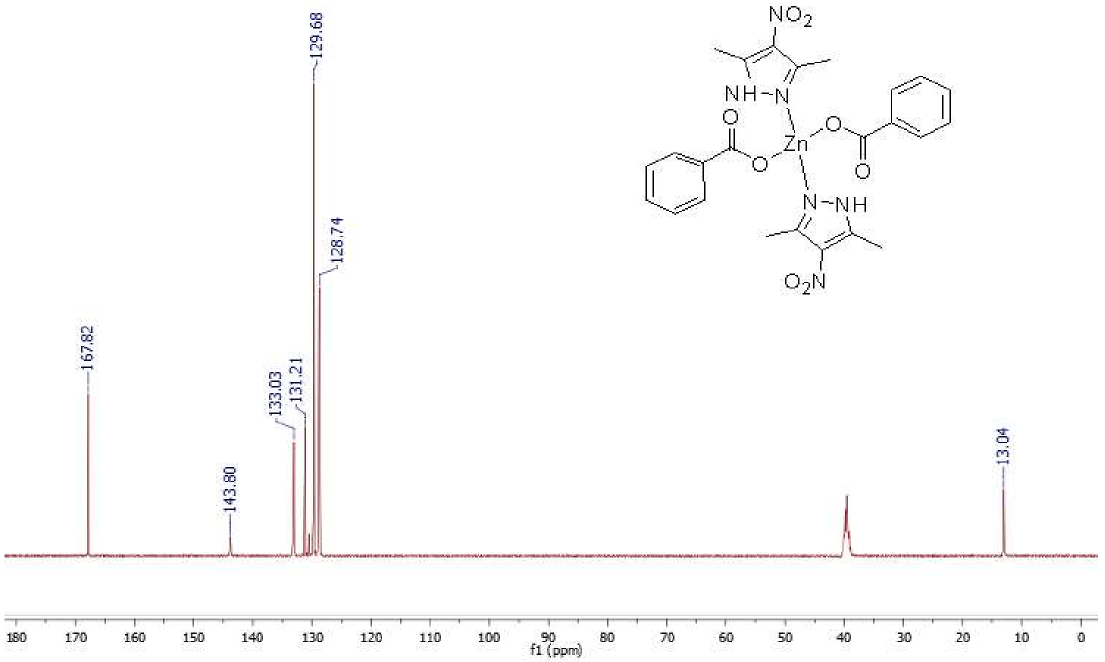

Figure S25. ^{13}C NMR spectrum of **5** in $(CD_3)_2SO$**.**

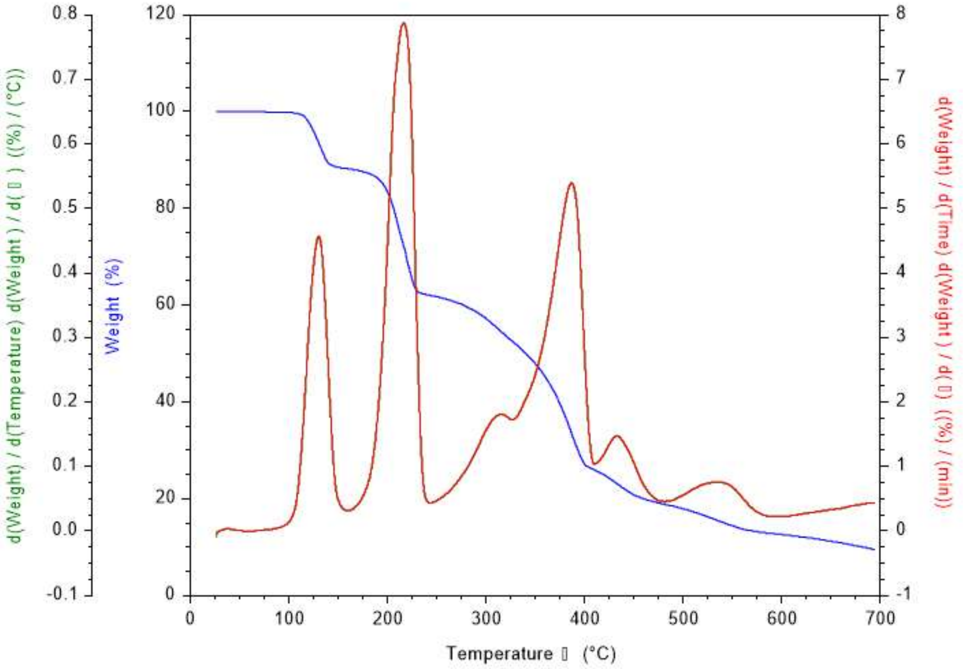

Figure S26. Thermogravimetric (TG) analysis and derivative thermogravimetric (DTG) of **1** in nitrogen atmosphere.

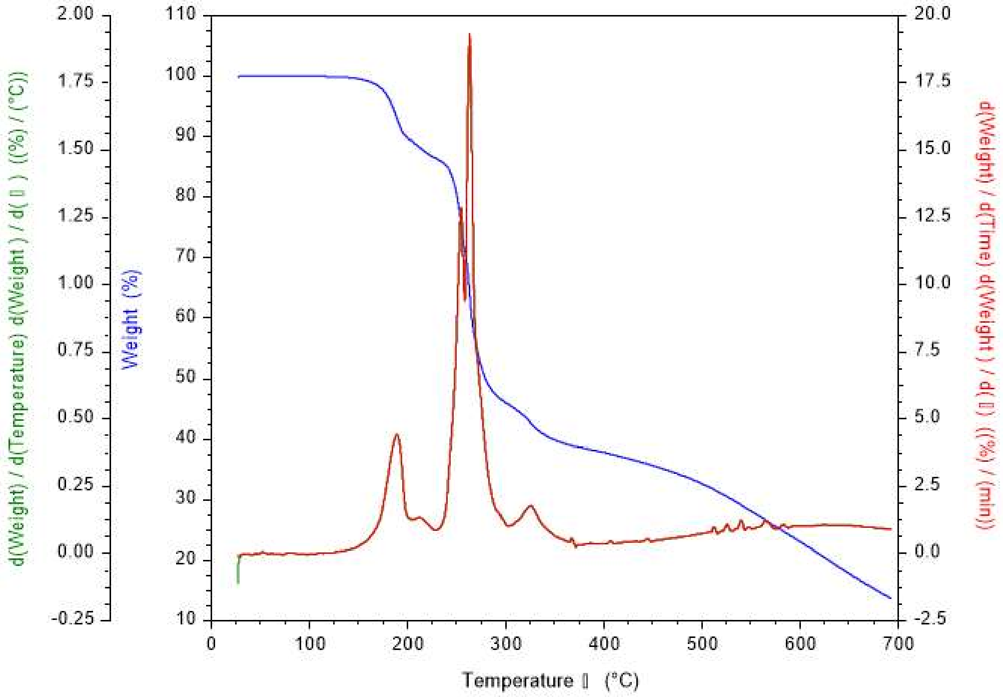

Figure S27. Thermogravimetric (TG) analysis and derivative thermogravimetric (DTG) of **2** in nitrogen atmosphere.

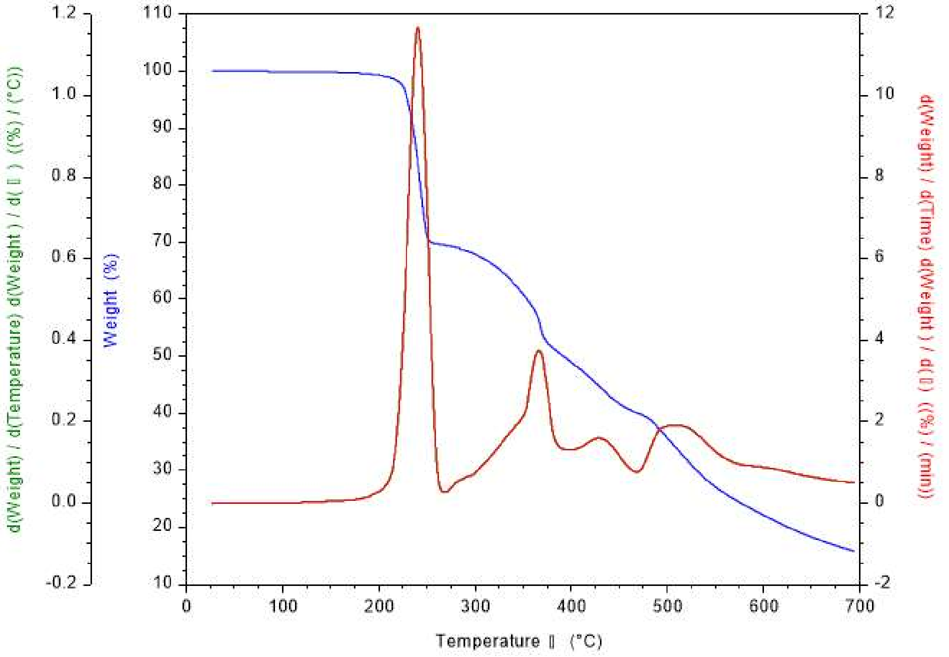

Figure S28. Thermogravimetric (TG) analysis and derivative thermogravimetric (DTG) of **3** in nitrogen atmosphere.

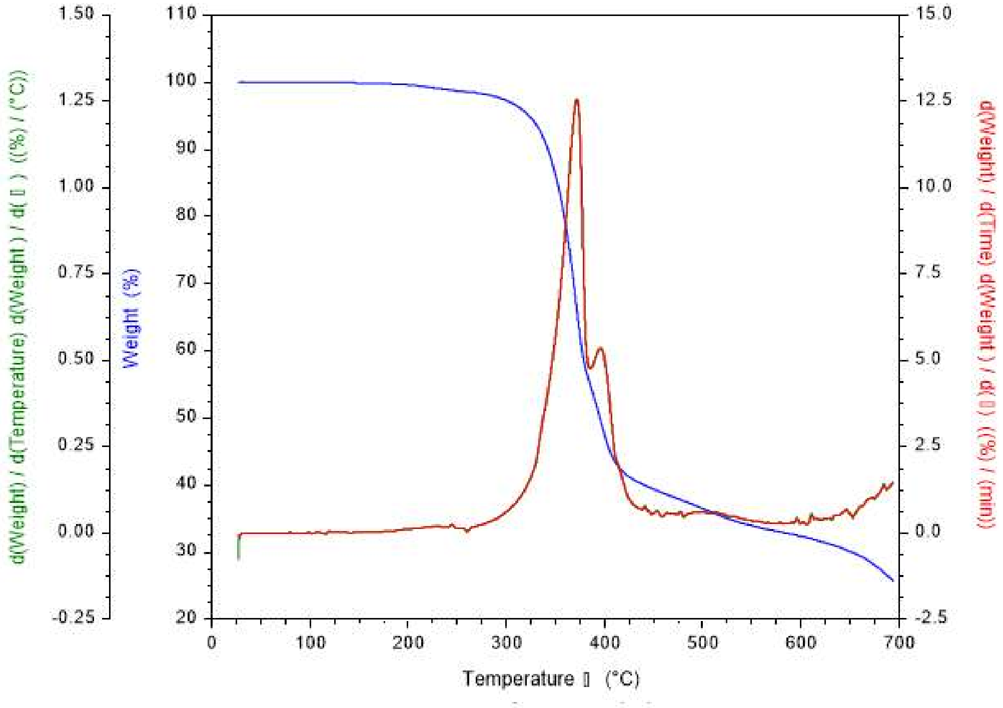

Figure S29. Thermogravimetric (TG) analysis and derivative thermogravimetric (DTG) of **5** in nitrogen atmosphere.

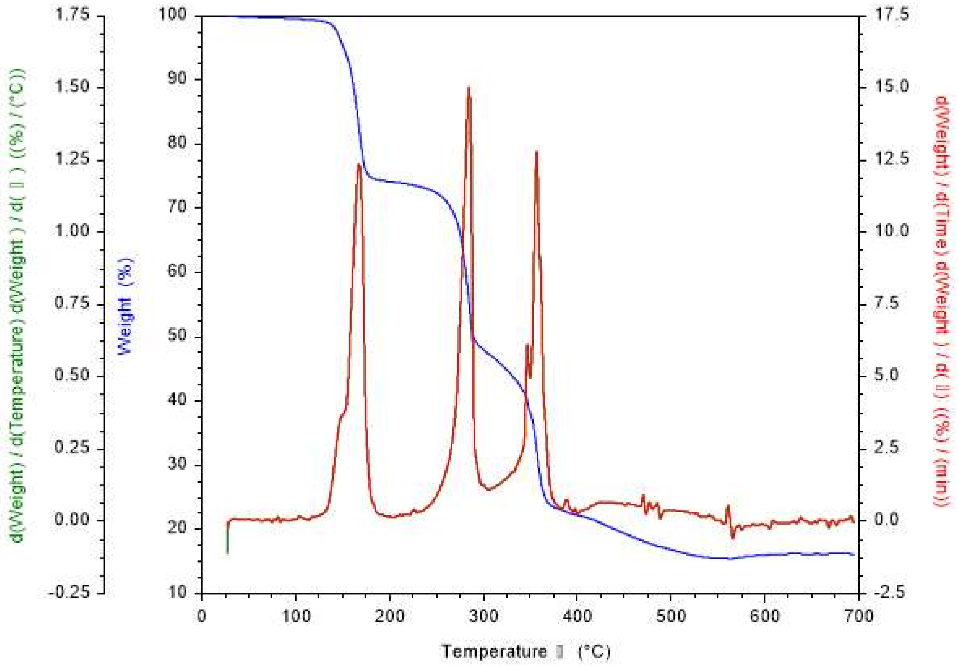

Figure S30. Thermogravimetric (TG) analysis and derivative thermogravimetric (DTG) of **6** in nitrogen atmosphere.

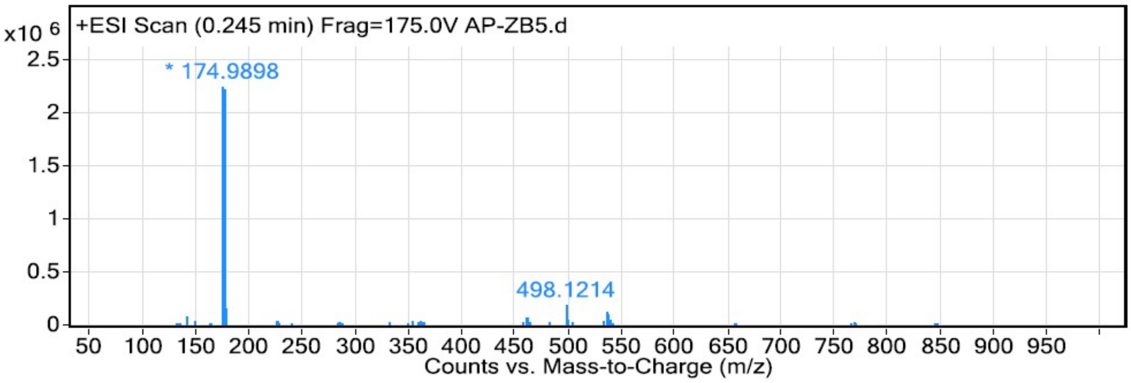

Figure S31. HRMS (ESI+) spectrum of **1** in acetonitrile

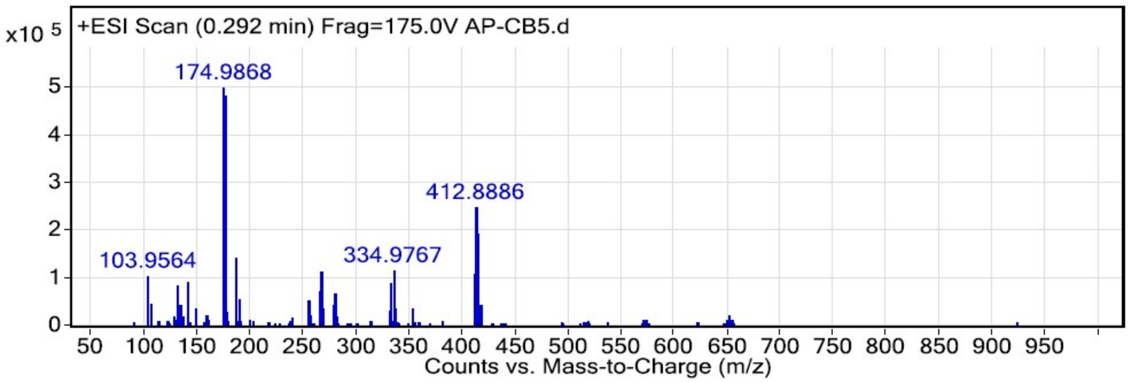

Figure S32. HRMS (ESI+) spectrum of **2** in acetonitrile**.**

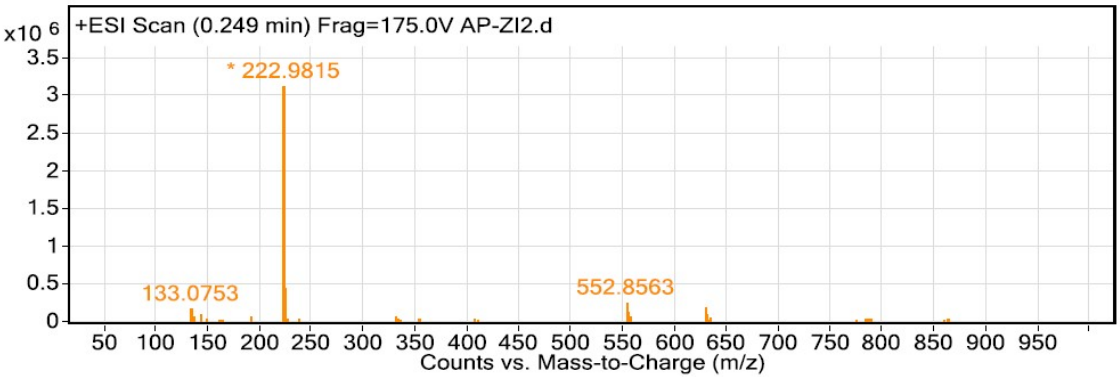

Figure S33. HRMS (ESI+) spectrum of **3** in acetonitrile**.**

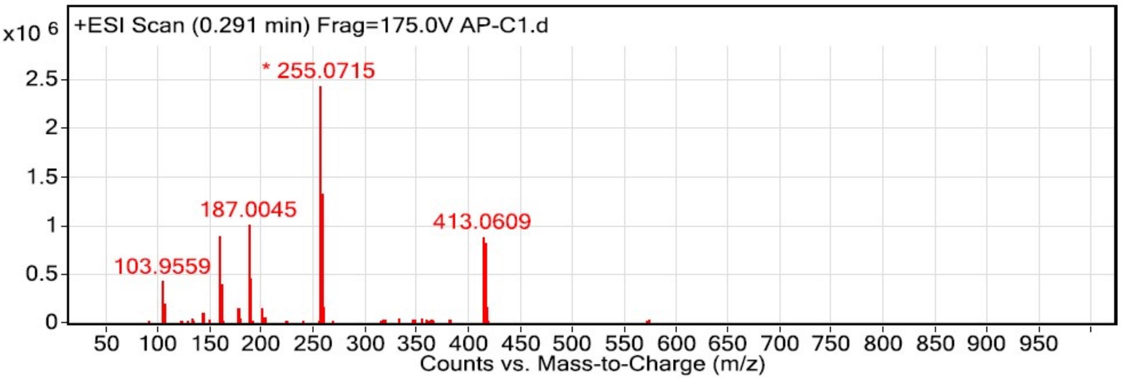

Figure S34. HRMS (ESI+) spectrum of **4** in acetonitrile**.**

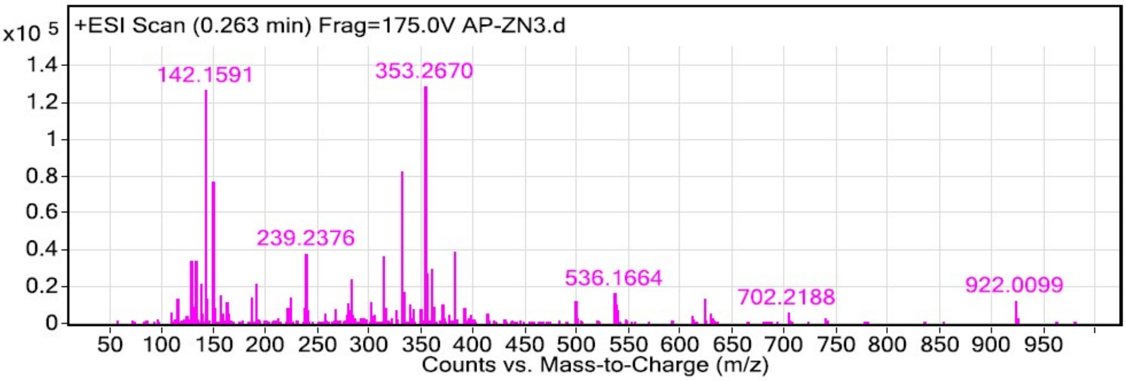

Figure S35. HRMS (ESI+) spectrum of **5** in acetonitrile**.**

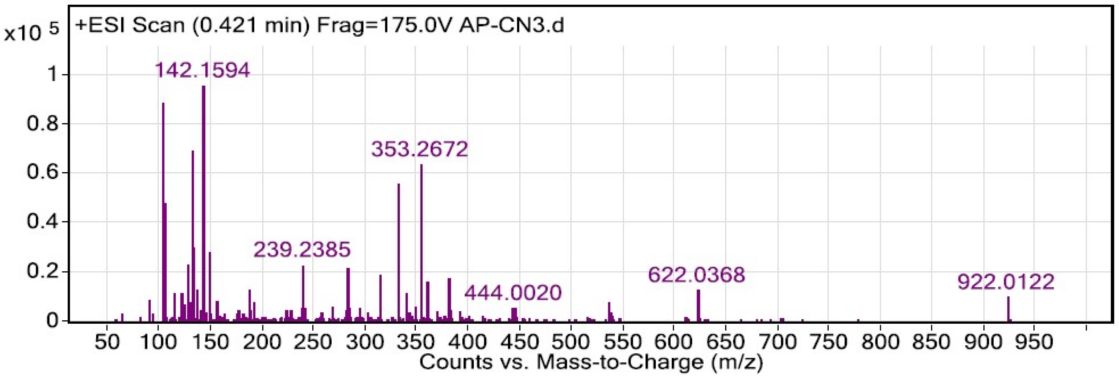

Figure S36. HRMS (ESI+) spectrum of **6** in acetonitrile**.**

2. Characterization of polymers

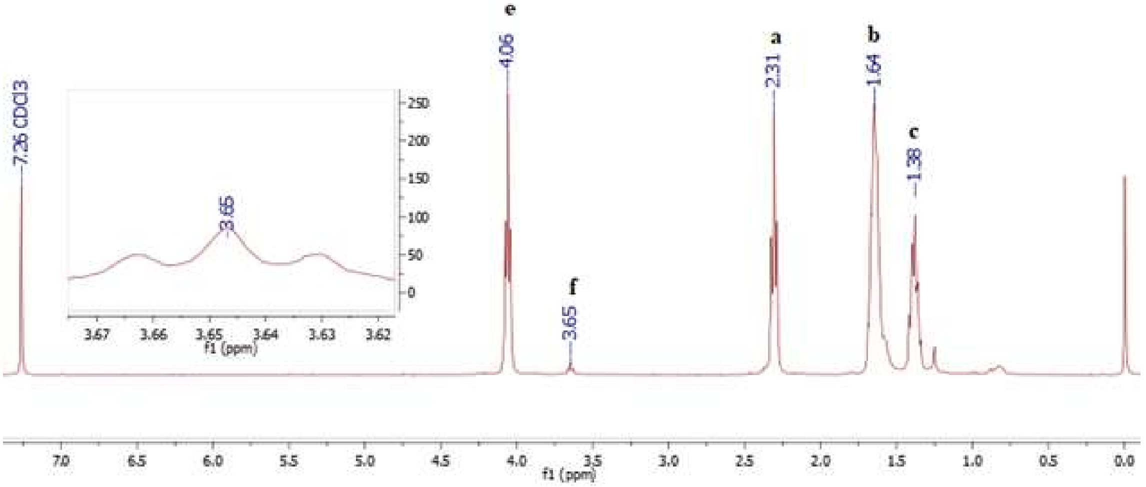

Figure S37. ^{1}H NMR spectrum of polymer obtained with initiator **1** in $CDCl_3$

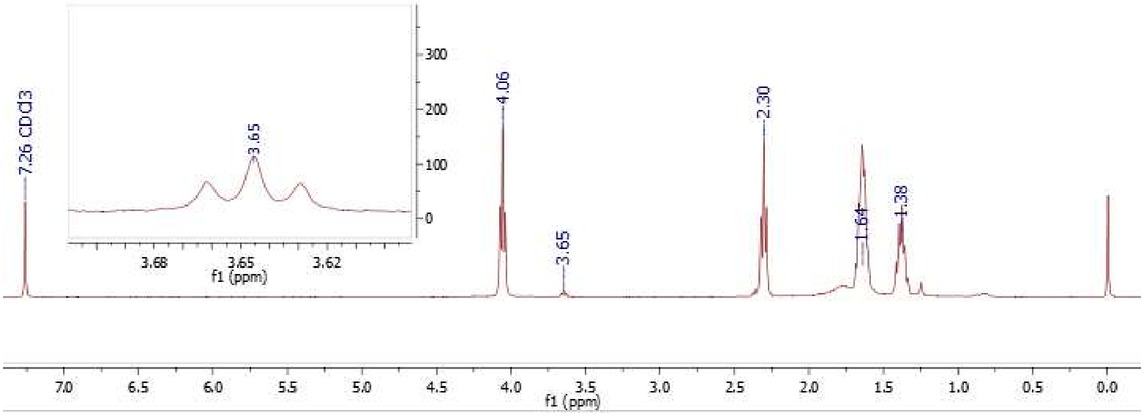

Figure S38. ^{1}H NMR spectrum of polymer obtained with initiator **2** in $CDCl_3$

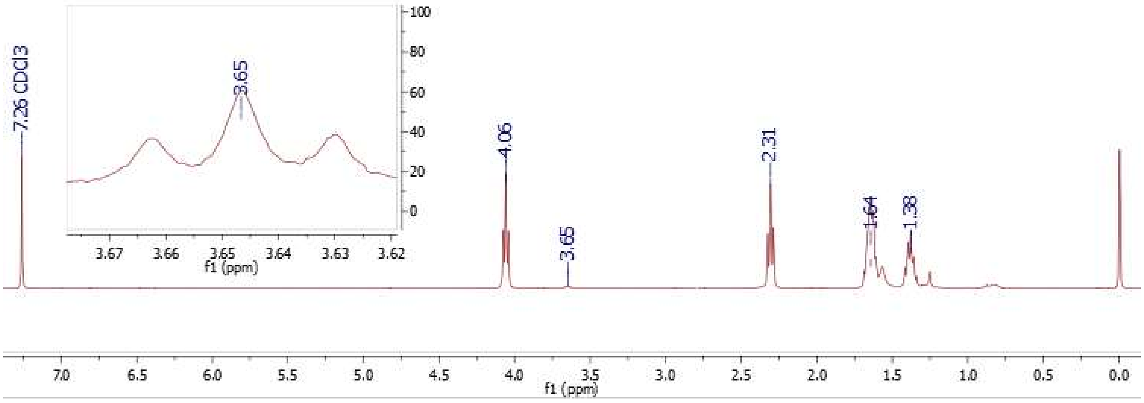

Figure S39. ^{1}H NMR spectrum of polymer obtained with initiator **3** in $CDCl_3$

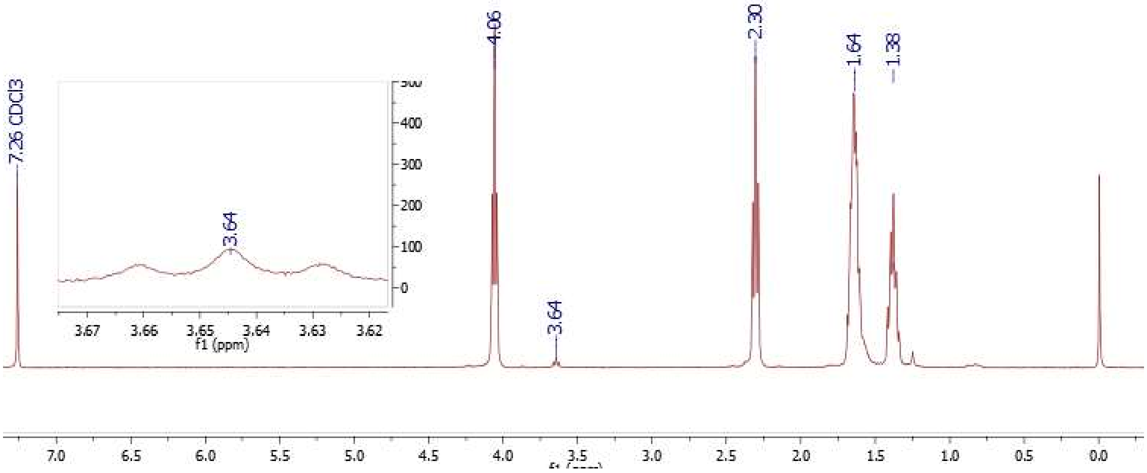

Figure S40. ^{1}H NMR spectrum of polymer obtained with initiator **4** in $CDCl_3$

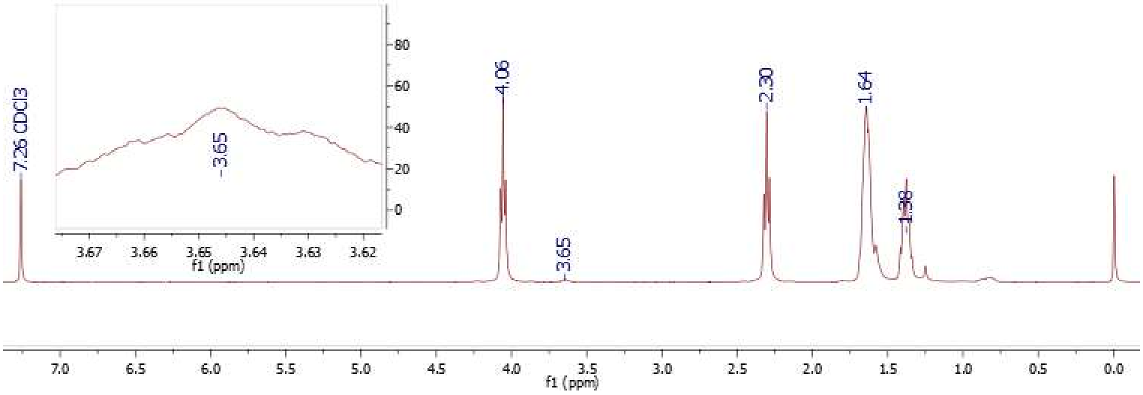

Figure S41. ^{1}H NMR spectrum of polymer obtained with initiator **5** in $CDCl_3$

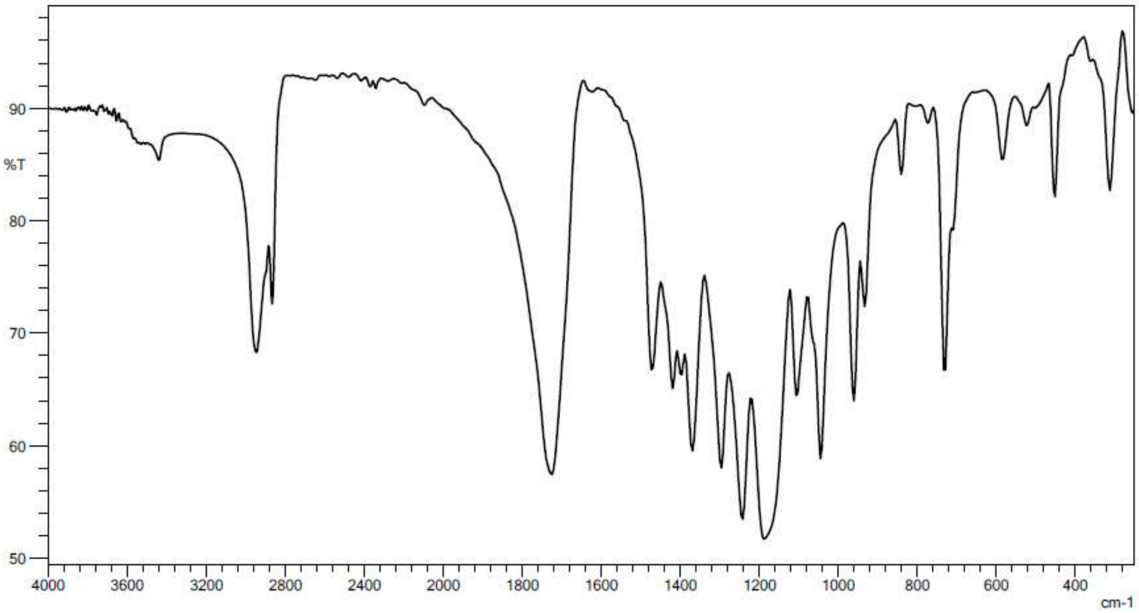

Figure S42. FT-IR (KBr) of polymer obtained with initiator **1**.

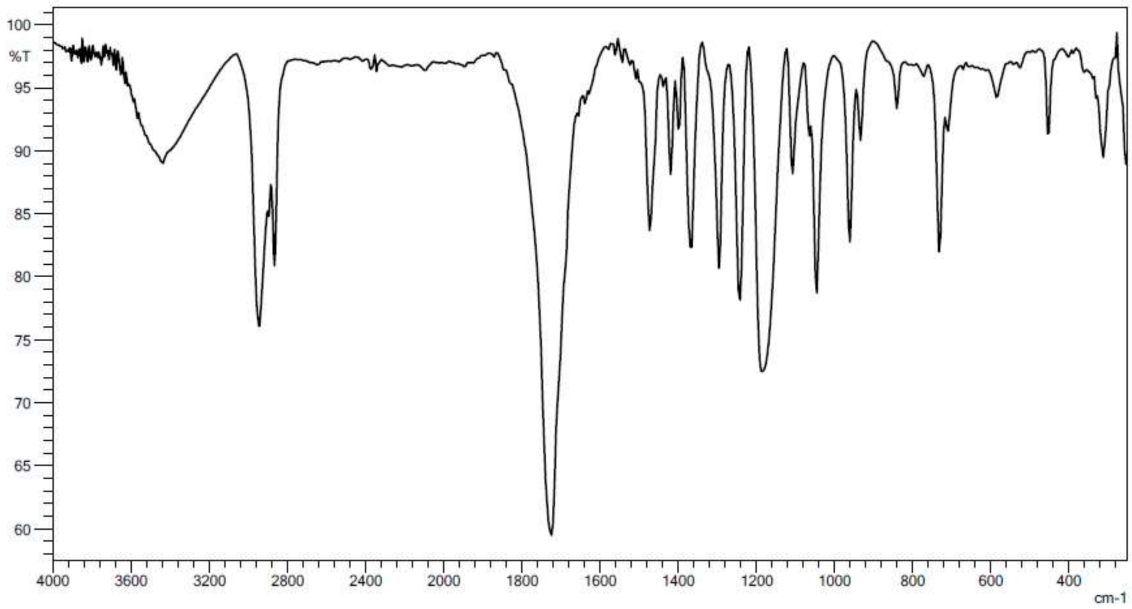

Figure S43. FT-IR (KBr) of polymer obtained with initiator **2**.

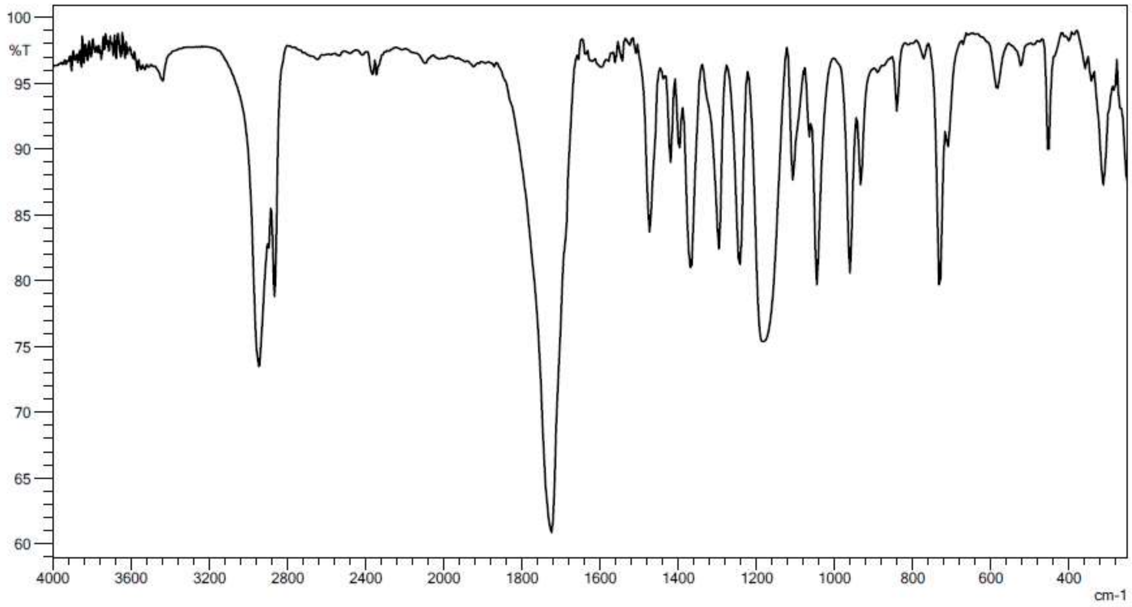

Figure S44. FT-IR (KBr) of polymer obtained with initiator **3**.

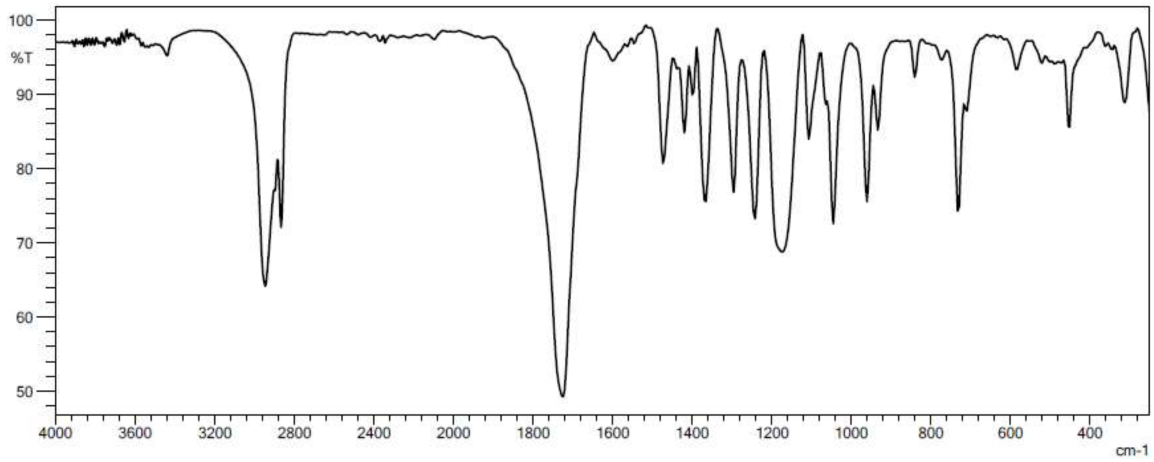

Figure S45. FT-IR (KBr) of polymer obtained with initiator **4**.

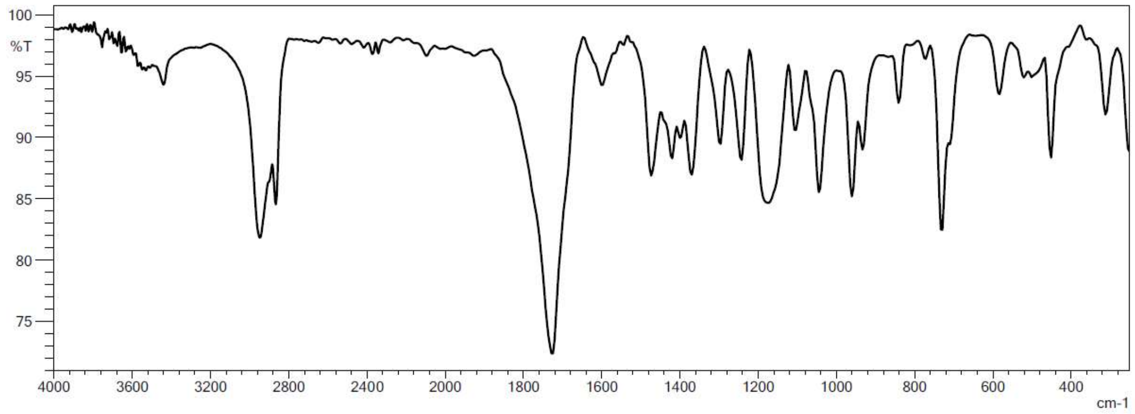

Figure S46. FT-IR (KBr) of polymer obtained with initiator **5**.

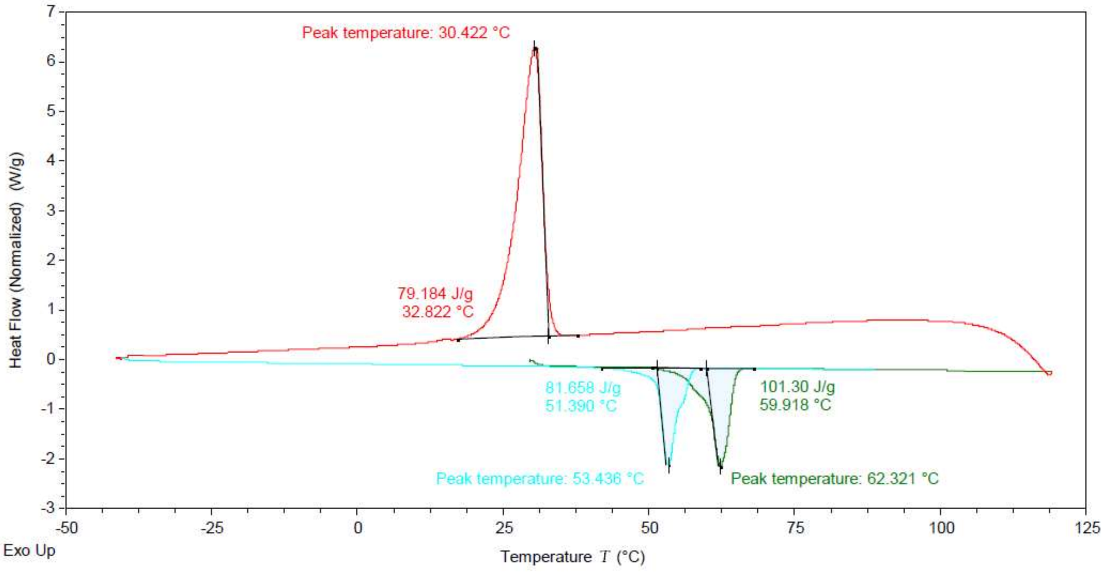

Figure S47. DSC of polymer obtained with initiator **1**

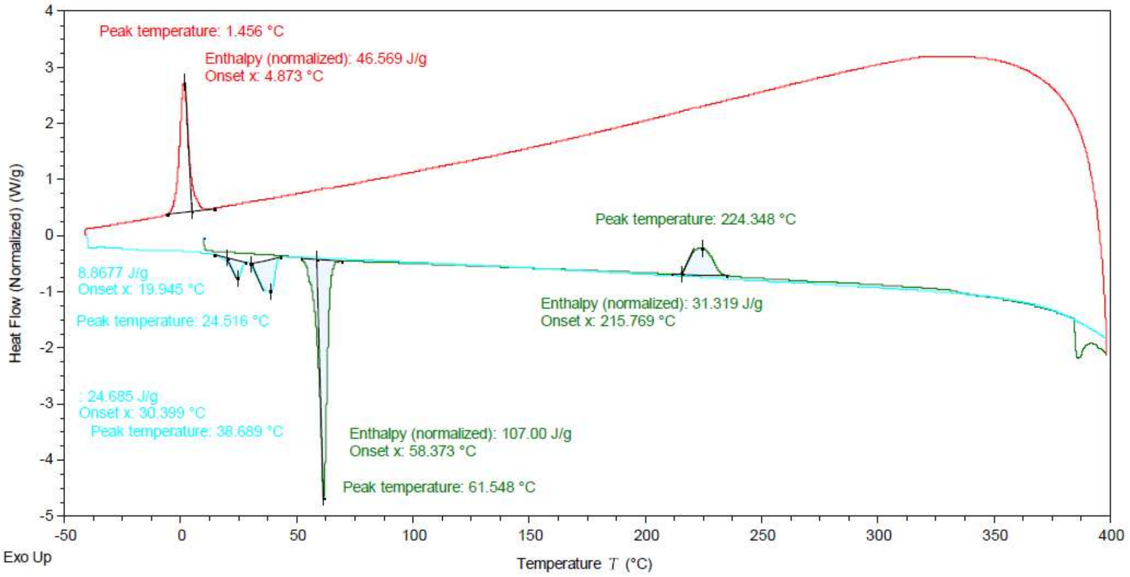

Figure S48. DSC of polymer obtained with initiator **2**

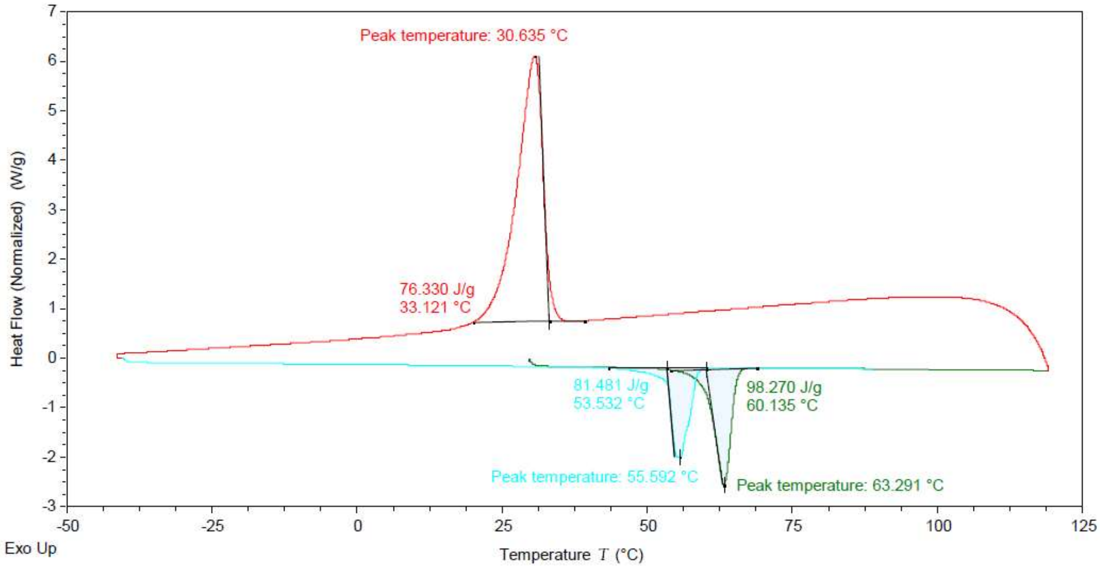

Figure S49. DSC of polymer obtained with initiator **3**

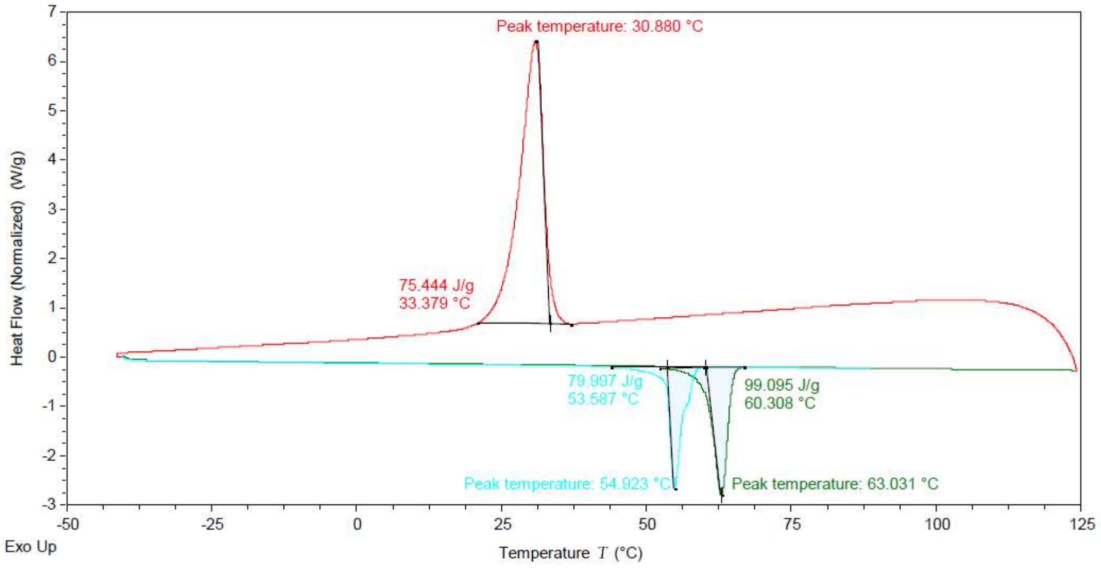

Figure S50. DSC of polymer obtained with initiator **4**

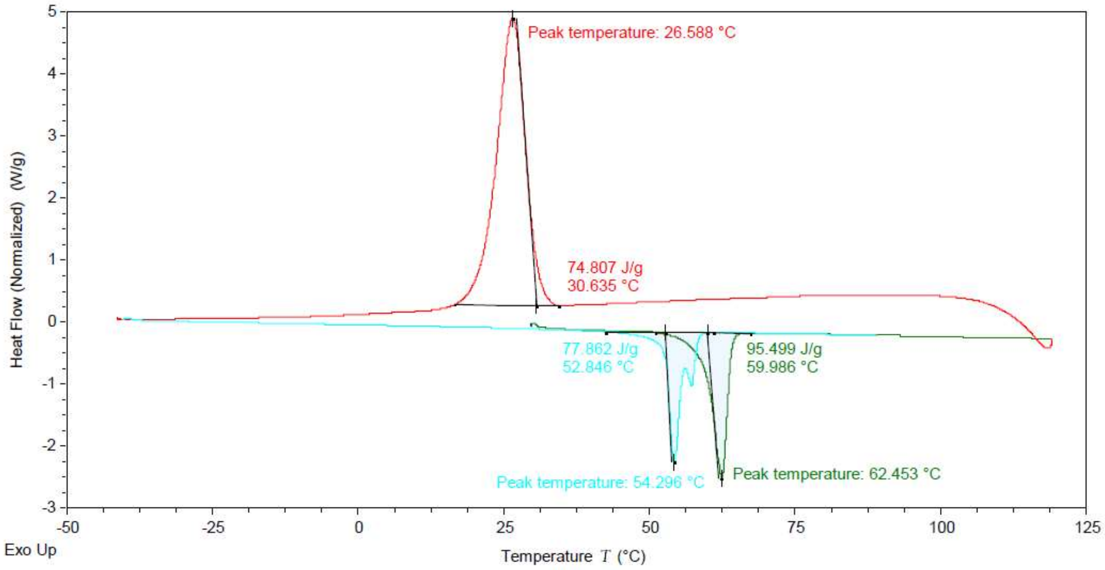

Figure S51. DSC of polymer obtained with initiator **5**

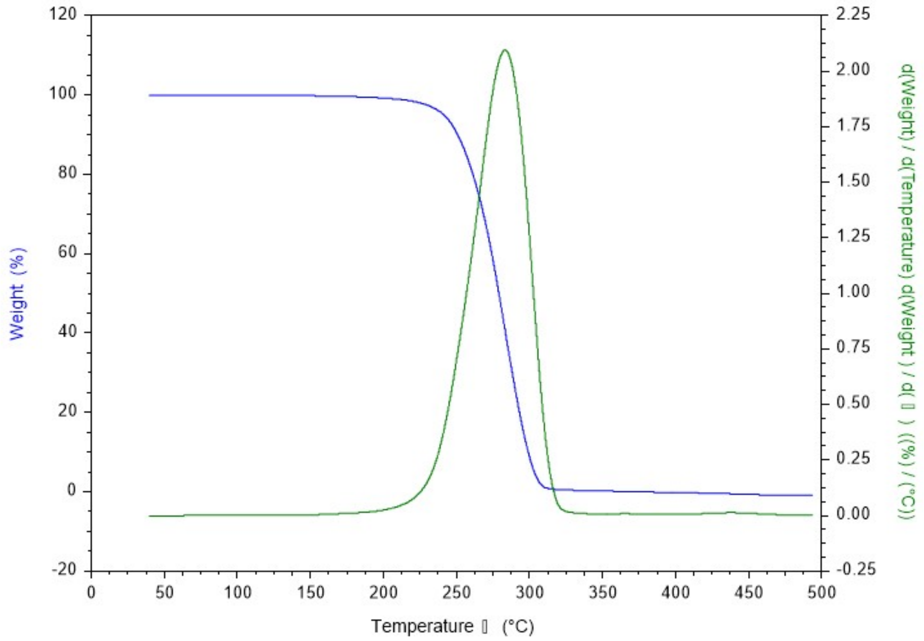

Figure S52. TGA of polymer obtained with initiator **1**

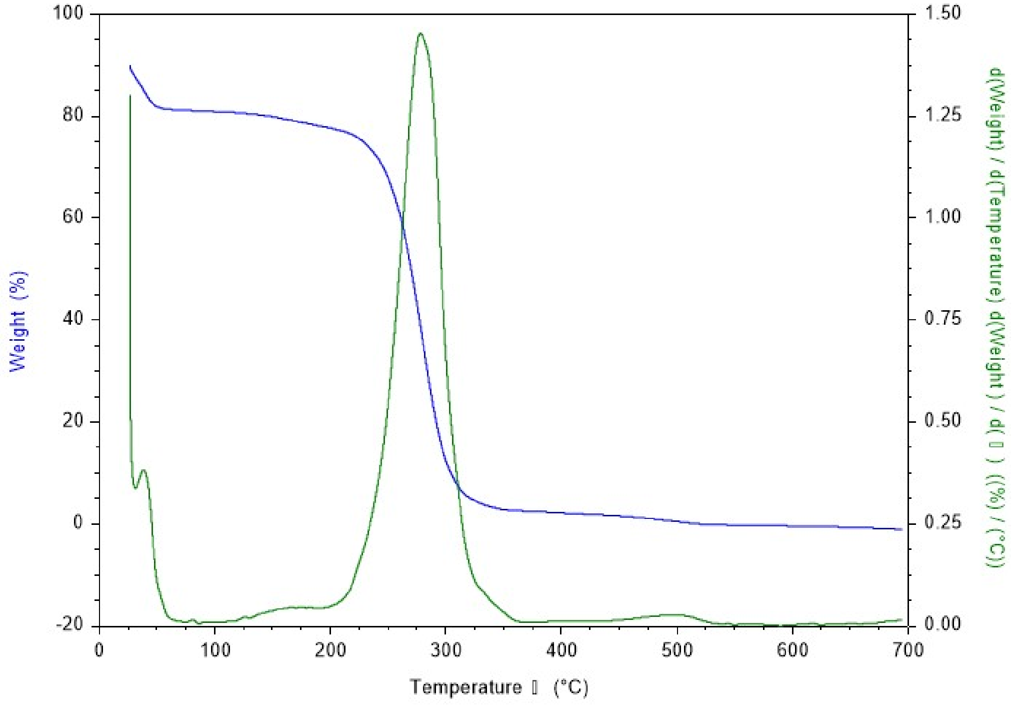

Figure S53. TGA of polymer obtained with initiator **2**

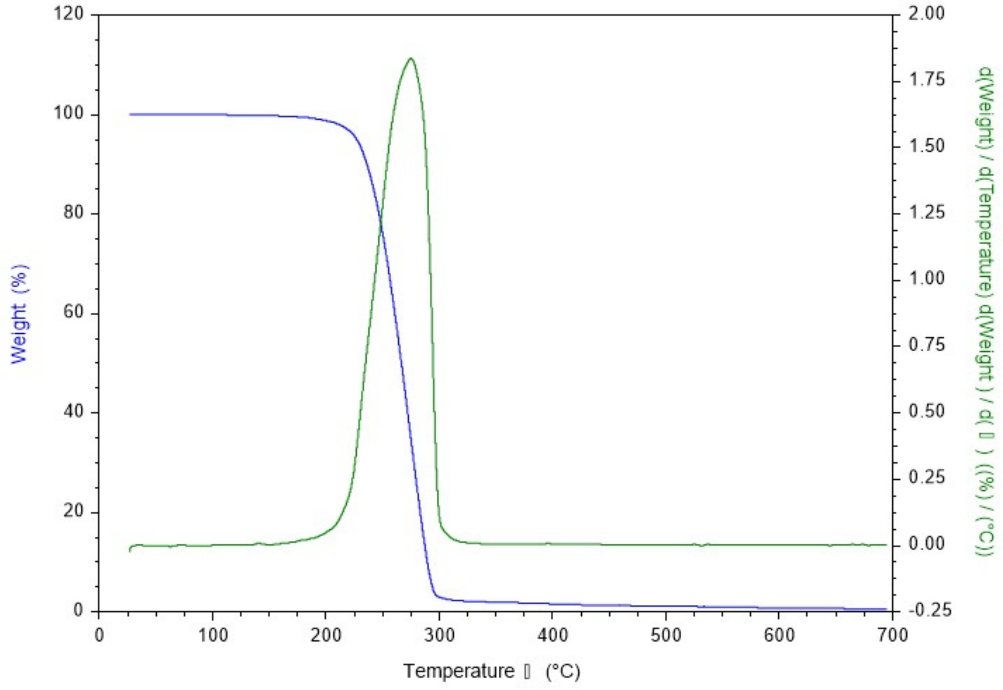

Figure S54. TGA of polymer obtained with initiator **4.**

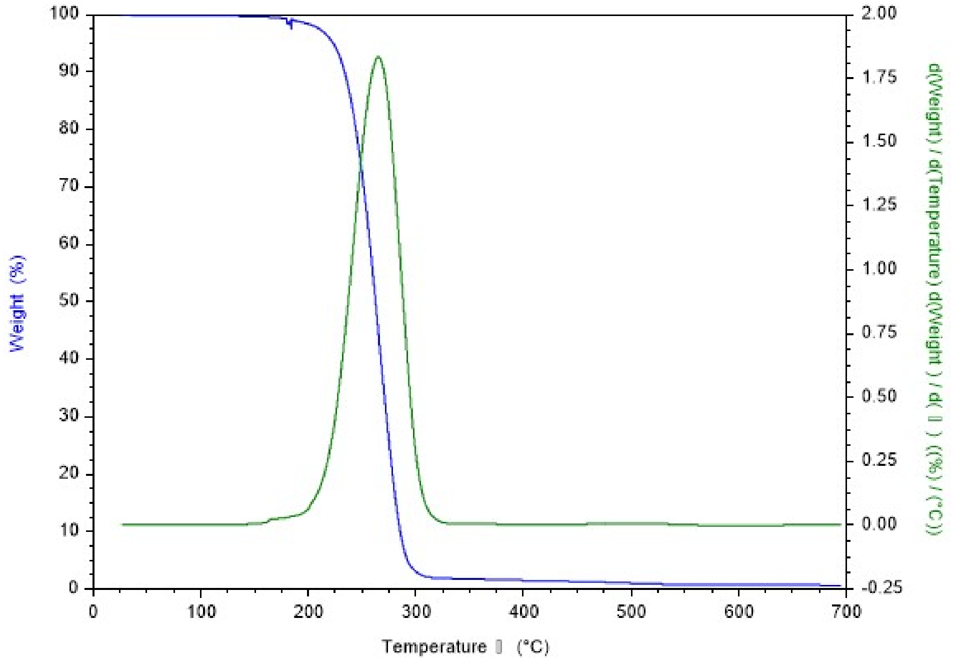

Figure S55. TGA of polymer obtained with initiator **5**

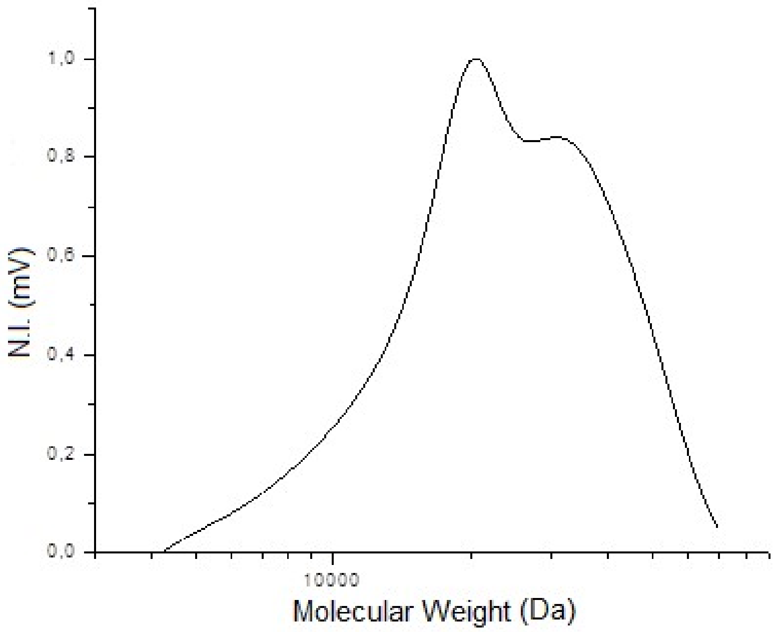

Figure S56 GPC curve of the polycaprolactone produced using **1**

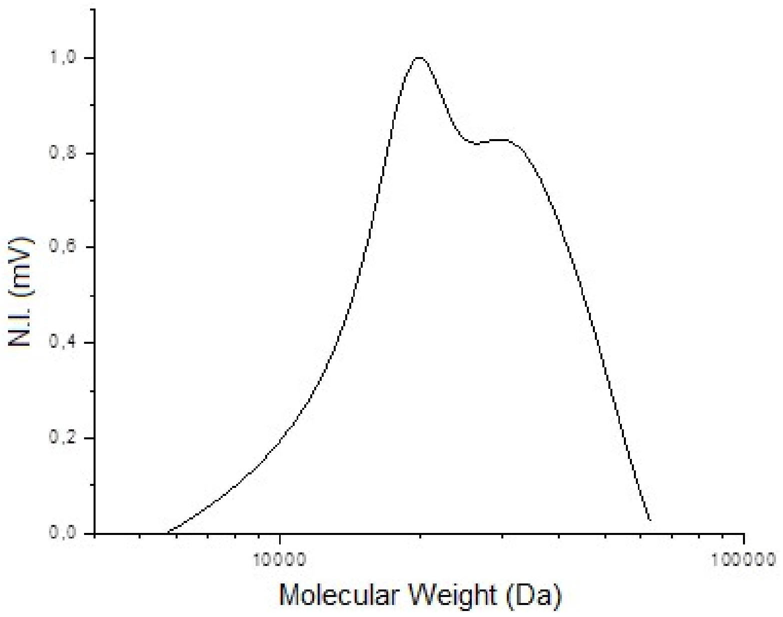

Figure S57. GPC curve of the polycaprolactone produced using **2**

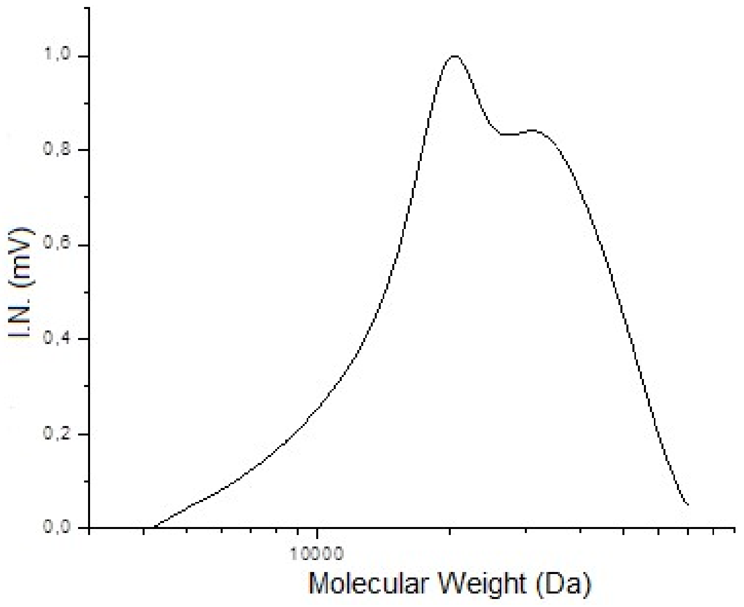

Figure S58. GPC curve of the polycaprolactone produced using **3**

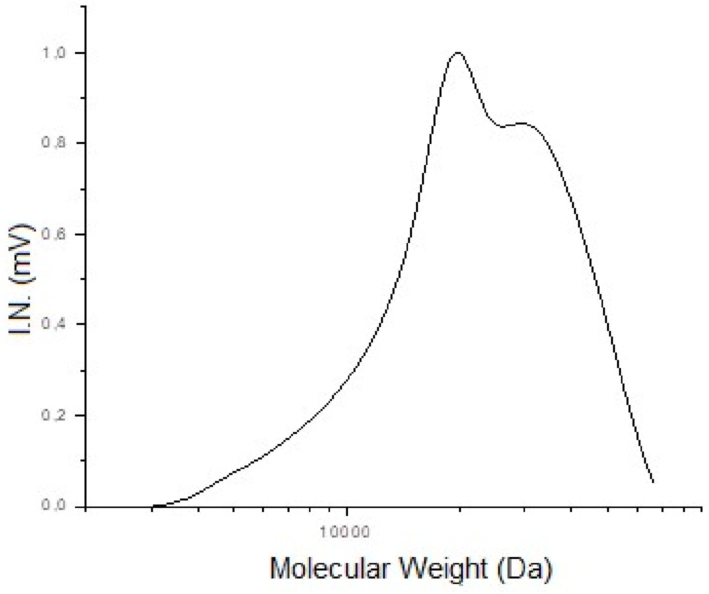

Figure S59. GPC curve of the polycaprolactone produced using **4**

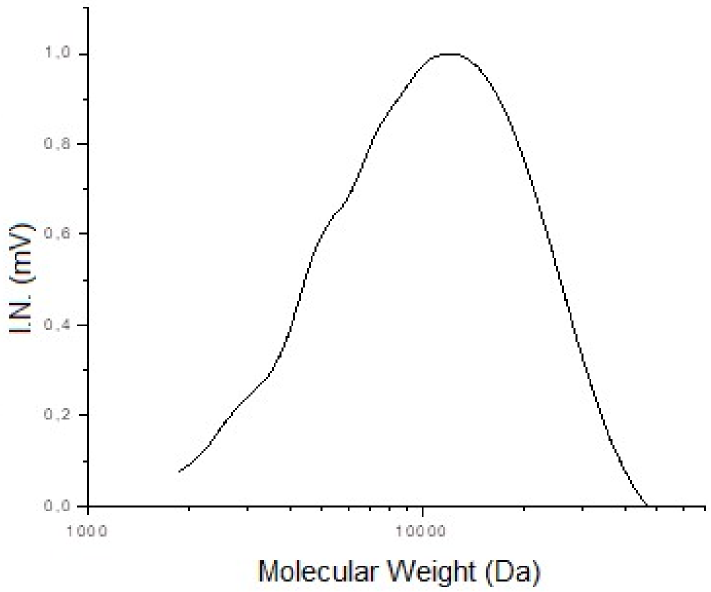

Figure S60. GPC curve of the polycaprolactone produced using **5**
